# Global, regional, and national burden and trends of intracerebral hemorrhage among adolescents and young adults aged 15–39 years from 1990 to 2021: a comprehensive trend analysis based on the global burden of disease study 2021

**DOI:** 10.3389/fneur.2025.1538413

**Published:** 2025-04-23

**Authors:** Xuanchen Liu, Xiaoxiong Yang, Yaoju Meng, Boyang Wen, Kun Yan, Qiyi Zhang, Junhao Wang, Yifei Su, Xiaochen Niu, Yingda Song, Rui Cheng, Hongming Ji, Guijun Jia, Chunhong Wang

**Affiliations:** ^1^The Neurosurgery Department of Shanxi Provincial People's Hospital, Shanxi Medical University, Taiyuan, China; ^2^Shanxi Provincial People's Hospital, Taiyuan, Shanxi, China; ^3^RWTH Aachen University, Aachen, Germany

**Keywords:** global burden of disease, intracerebral hemorrhage, adolescents and young adults, trend analysis, social development index

## Abstract

**Background:**

Intracerebral hemorrhage (ICH) poses a significant health challenge, notably affecting adolescents and young adults (AYAs) aged 15–39 years. Despite advancements in medical technology, the global burden of ICH remains substantial, influenced by lifestyle factors, socioeconomic conditions, and disparities in healthcare access. This study leverages data from the 2021 Global Burden of Disease (GBD) to conduct a comprehensive analysis of ICH trends and burdens at global, regional, and national levels, emphasizing the role of the Social Development Index (SDI).

**Methods:**

We utilized data from the GBD 2021 to assess the incidence, prevalence, mortality, and disability-adjusted life years (DALYs) associated with ICH from 1990 to 2021, covering 204 countries and regions. Age-standardized rates were calculated to ensure comparability. Temporal trends were evaluated using Joinpoint regression analysis, and future projections were made using a Bayesian Age-Period-Cohort model.

**Results:**

In 2021, ~246,938 new cases of ICH were reported among the global AYAs population, reflecting a decline in age-standardized incidence rates from 11.85 per 100,000 in 1990 to 8.14 in 2021. Prevalence rates also decreased from 124.44 to 94.58 per 100,000. Mortality rates and DALYs exhibited similar downward trends. Significant regional disparities were observed, with high SDI regions experiencing lower ICH burdens than low SDI regions, highlighting the influence of socioeconomic factors and healthcare access.

**Conclusion:**

While the global burden of ICH among AYAs has declined, significant disparities persist, particularly in regions with lower SDI. To further mitigate the impact of ICH, public health initiatives should focus on enhancing healthcare infrastructure, promoting health education, and addressing socioeconomic inequalities.

## 1 Introduction

Over recent decades, the global incidence and burden of intracerebral hemorrhage (ICH) have undergone significant transformations, most notably impacting adolescents and young adults (AYAs) within the age range of 15 to 39 years. As a leading contributor to stroke-related mortality and dependency, ICH poses substantial challenges to individual health and public health systems alike. Despite considerable advancements in healthcare infrastructure, chronic disease management, and acute ICH treatment on a global scale, the burden of ICH within the AYAs demographic remains intricately influenced by specific factors, including lifestyle, social roles, and health behaviors (1, 2).

Utilizing data from the Global Burden of Disease Study 2021 (GBD 2021), this study presents a detailed examination of the burden and trends associated with intracerebral hemorrhage (ICH) at global, regional, and national levels from 1990 to 2021 (3). The main aim of this study is to elucidate the epidemiological features and temporal trends of intracerebral hemorrhage in different regions and countries, stratified by varying levels of the Social Development Index (SDI). Specifically, this research examines the roles and impacts of socioeconomic determinants, lifestyle behaviors, and healthcare systems on the ICH burden within AYAs populations (4).

Through a comprehensive analysis of data from 204 countries, this study elucidates significant regional and inter-country disparities in the incidence and mortality rates of intracerebral hemorrhage. Particularly, the higher incidence and mortality rates observed in low- and middle-income countries are likely due to a combination of factors such as insufficient public health awareness, inadequate disease prevention strategies, and limited access to healthcare resources. Contrastingly, some high-income countries show enhanced access to hypertension screening, emergency medical services, and cutting-edge medical technologies targeted at AYAs (5, 6). However, the suboptimal healthcare infrastructure, deficient medical facilities, and inadequate health education prevalent in lower-income countries substantially impede the early management of ICH and the prevention of associated chronic diseases (1).

This study utilizes a Bayesian Age-Period-Cohort model for predicting trends in the incidence of ICH over the forthcoming two decades. By leveraging this model, the research aims to furnish empirical data that can inform public health policies, thereby maximizing resource utilization, promoting health education, and improving chronic disease management among AYAs. These strategies are pivotal for mitigating the long-term consequences of ICH and advancing global health equity. Furthermore, the study underscores the necessity of enhancing data quality, deepening the analysis of urban-rural and socioeconomic disparities, and incorporating dynamic social and cultural variables. Such efforts are essential for comprehensively understanding the impact and evolving trends of ICH within AYAs populations, thereby providing a robust foundation for developing more effective public health guidelines and ultimately improving the health and quality of life for these cohorts.

## 2 Methods

### 2.1 Data source

The current study utilized data from the GBD 2021, with a particular focus on the incidence of ICH in the AYAs population, specifically those aged 15 to 39 years. We downloaded data covering 204 countries and 21 regions, allowing for a comprehensive global and regional analysis of the ICH burden.

ICH was identified using ICD-10 code I61 (non-traumatic intracerebral hemorrhage) and ICD-9 code 431. These codes include hemorrhages within the brain parenchyma, typically due to hypertensive arteriopathy or cerebral amyloid angiopathy (7).

The GBD 2021 dataset provided age-standardized incidence rates (ASIR), prevalence rates (ASPR), mortality rates (ASMR), and disability-adjusted life years (DALYs) for ICH. Data from 1990 to 2021 were analyzed to assess global and regional trends, focusing on differences by age, sex, and SDI (8).

### 2.2 Age standardization

To maintain the uniformity of ICH burden across populations with varying age structures, we used age standardization through the direct method. Age-standardized rates (ASR) for incidence, prevalence, mortality, and disability-adjusted life years (DALYs) were calculated based on the GBD standard population. This method accounts for differences in age distribution, allowing for meaningful comparisons across regions and periods. The ASR is calculated by determining a weighted average of age-specific rates, where each age-specific rate is adjusted according to the population size of the corresponding age group in the standard population. This approach provides an estimate of what the rates would be if all populations had the same age structure, thereby facilitating accurate comparisons of ICH burden across countries and regions.

### 2.3 Joinpoint analysis

We employed Joinpoint regression analysis to examine temporal trends in the incidence, prevalence, mortality, and DALYs of ICH in the AYAs population between 1990 and 2021. Joinpoint analysis identifies points where significant changes in trend occur, referred to as “joinpoints.” For each segment between joinpoints, The Annual Percentage Change (APC) was determined to measure the yearly variation in the age-standardized rate (ASR). The APC estimates the trend's direction and magnitude, with 95% confidence intervals (CI) indicating statistical significance when they do not include zero (9).

In addition, The Average Annual Percentage Change (AAPC) was calculated to provide an overview of the overall trend throughout the entire study period. The AAPC is a weighted average of the APCs across all segments, providing a single indicator of the long-term trend. Both APC and AAPC are reported with 95% CIs, and trends were considered statistically significant with a *p* < 0.05.

The Joinpoint Regression Program (version 4.9.0.0) was used for this analysis, allowing us to identify key periods of change in ICH burden among the AYAs population and assess the overall trajectory of disease trends.

### 2.4 Cross-country social inequalities analysis

Using the slope index of inequality (SII) and health inequality concentration index (CI) to assess the unequal distribution of burden on young patients aged 15–39 with cerebral hemorrhage in various countries (10). The SII was calculated by regressing country-level ASPR and ASDR on a scale of income-related relative social position (11). The CI is calculated by integrating the area under the Lorenz concentration curve. Higher absolute RCI values signify greater inequality.

### 2.5 Bayesian age-period-cohort prediction

To forecast the incidence of cerebral hemorrhage in young adults (15–39 years old) in the future, a Bayesian Age Period Queue (BAPC) model was employed to evaluate and project the number and prevalence of this disease from 2021 to 2040, and ASR was calculated based on gender. The BAPC model utilizes Integrated Nested Laplace Approximations (INLA) to estimate marginal posterior distributions, thereby addressing some of the mixing and convergence challenges associated with traditional Markov Chain Monte Carlo (MCMC) sampling in Bayesian inference. Analyses were conducted using the BAPC and INLA packages available in the R statistical software environment (12, 13).

### 2.6 Statistics

All statistical analyses in this study were conducted using R software (version 4.2.2), and a *p-*value of < 0.05 was considered statistically significant.

This study utilized data exclusively obtained from the GBD 2021 database, a publicly available resource managed by the Institute for Health Metrics and Evaluation (IHME). The data utilized in this research are anonymized and aggregated and do not contain any personally identifiable information. As such, this study meets the criteria for exemption from full ethical review according to the guidelines provided by the Shanxi Provincial People's Hospital Ethics Committee and the University of Washington's IRB, which has oversight over the GBD project.

## 3 Results

### 3.1 Global trends

The GBD report for 2021 indicates that there were 246,938.25 new cases of ICH diagnosed among AYAs aged 15–39 years, representing a slight decrease compared to 247,927.77 cases in 1990. From 1990 to 2021, the ASIR for the condition decreased from 11.85 [95% Uncertainty Interval (UI): 7.96–16.69] per 100,000 individuals to 8.14 (95% UI: 5.65–11.31). Despite the reduction in ASIR, the total number of prevalent cases showed a modest increase, reaching 2,853,528.34 by 2021. The ASPR decreased from 124.44 (UI: 104.42–147.40) per 100,000 inhabitants in 1990 to 94.58 (UI: 81.56–109.39) in 2021. In 2021, the number of deaths attributed to ICH was 85,038.37, representing a decrease from ½89,160.93 deaths recorded in 1990. The ASDR exhibited a significant decline from 4.30 (UI: 3.91–4.66) per 100,000 population in 1990 to 2.79 (UI: 2.51–3.10) per 100,000 population in 2021. The total DALYs lost due to ICH decreased from 5,673,698.79 in 1990 to 5,385,247.12 in 2021. The age-standardized DALYs rate also declined from 271.00 (UI: 246.98–293.66) per 100,000 population in 1990 to 177.45 (UI: 159.88–196.14) in 2021. Consistently, males experienced a higher disease burden compared to females, with higher ASRs for incidence, prevalence, deaths, and DALYs among males compared to females (Table 1 and Supplementary Table 1).

**Table 1:** Global incidence, prevalence, DALYs, and deaths of ICH in AYAs in 2021.

**Characteristics**		**Incidence**			**Prevalence**			**DALYs**			**Deathes**		
		**Num_2021 (95% UI)**	**ASR_2021 (95% UI)**	**AAPCs 1990–2021 (95% CI)**	**Num_2021 (95% UI)**	**ASR_2021 (95% UI)**	**AAPCs 1990–2021 (95% CI)**	**Num_2021 (95% UI)**	**ASR_2021 (95% UI)**	**AAPCs 1990–2021 (95% CI)**	**Num_2021 (95% UI)**	**ASR_2021 (95% UI)**	**AAPCs 1990–2021 (95% CI)**
Global	Global	246938.25 (171761.43–342183.38)	8.14 (5.65–11.31)	−1.24 (−1.31–1.16)	2853528.34 (2462066.44–3300117.99)	94.57 (81.56–109.39)	−0.90 (−0.94–0.87)	5385247.12 (4854154.83–5951021.00)	177.45 (159.88–196.14)	−1.37 (−1.59–1.15)	85038.37 (76402.76–94272.88)	2.79 (2.51–3.10)	−1.40 (−1.59–1.21)
Sex	Male	150829.75 (105911.05–207755.79)	9.81 (6.88–13.53)	−0.91 (−0.97–0.86)	1493809.68 (1289249.99–1725612.44)	97.65 (84.26–112.81)	−0.71 (−0.75–0.68)	3404908.82 (3023239.26–3825577.12)	221.23 (196.34–248.61)	−1.05 (−1.28–0.81)	55164.97 (48481.03–62138.34)	3.57 (3.14–4.03)	−1.06 (−1.29–0.82)
	Female	96108.50 (63554.63–137922.99)	6.44 (4.25–9.27)	−1.68 (−1.75–1.61)	1359718.66 (1169416.57–1574996.53)	91.45 (78.59–105.95)	−1.10 (−1.14–1.06)	1980338.30 (1732165.66–2238181.73)	132.71 (116.02–150.05)	−1.80 (−1.93–1.67)	29873.40 (26090.42–34203.82)	1.99 (1.74–2.28)	−1.91 (−2.05–1.78)
Sociodemographic index	High SDI	15186.60 (9514.19–22882.88)	4.02 (2.47–6.16)	−1.45 (−1.51–1.39)	286145.42 (244723.11–333083.15)	76.99 (65.57–89.80)	−0.80 (−0.82–0.79)	229305.32 (203630.18–259198.72)	58.92 (52.24–66.72)	−1.88 (−2.05–1.71)	3271.76 (2917.55–3741.77)	0.82 (0.73–0.94)	−2.05 (−2.24–1.86)
	High-middle SDI	38254.33 (26186.17–53538.56)	7.79 (5.27–11.05)	−1.57 (−1.67–1.47)	456335.02 (392061.39–531523.15)	95.58 (81.92–111.31)	−1.11 (−1.15–1.07)	760725.89 (672547.29–858863.68)	151.91 (134.23–171.46)	−1.74 (−2.22–1.25)	11988.80 (10523.74–13643.72)	2.34 (2.06–2.67)	−1.76 (−2.27–1.25)
	Middle SDI	82822.00 (57336.41–114710.85)	8.54 (5.88–11.88)	−1.44 (−1.48–1.41)	934987.71 (794379.52–1096730.24)	97.40 (82.67–114.29)	−1.10 (−1.15–1.04)	1865296.44 (1682393.93–2079862.63)	191.39 (172.58–213.42)	−1.62 (−1.77–1.47)	29754.24 (26825.91–33357.01)	3.03 (2.73–3.40)	−1.65 (−1.81–1.50)
	Low-middle-SDI	71091.25 (49690.56–98034.29)	9.15 (6.41–12.58)	−1.04 (−1.10–0.99)	740689.40 (639583.86–852643.81)	94.46 (81.63–108.69)	−0.63 (−0.65–0.61)	1592446.54 (1351763.50–1854835.62)	204.74 (173.89–238.42)	−1.48 (−1.60–1.36)	25367.47 (21323.69–29793.69)	3.28 (2.76–3.85)	−1.52 (−1.65–1.39)
	Low SDI	39396.01 (28448.66–53076.91)	9.68 (7.04–12.94)	−1.42 (−1.48–1.35)	433199.92 (385868.27–483455.73)	103.58 (92.56–115.39)	−0.89 (−0.92–0.86)	932365.65 (749523.09–1122113.25)	226.85 (182.83–272.72)	−1.31 (−1.50–1.13)	14574.15 (11573.57–17667.17)	3.62 (2.88–4.38)	−1.34 (−1.51–1.17)
Regions	Andean Latin America	1588.64 (1116.37–2182.64)	5.88 (4.13–8.08)	−2.27 (−2.36–2.18)	22694.08 (20337.82–25172.14)	83.79 (75.09–92.94)	−1.39 (−1.43–1.35)	34350.92 (26361.95–43882.28)	127.13 (97.57–162.38)	−2.85 (−3.61–2.09)	536.81 (403.37–691.39)	1.99 (1.50–2.56)	−2.92 (−3.72–2.12)
	Australasia	227.25 (118.50–386.28)	2.10 (1.07–3.61)	−1.64 (−1.75–1.54)	4935.62 (4076.50–5880.53)	45.37 (37.37–54.18)	−0.68 (−0.75–0.61)	2097.12 (1758.46–2495.87)	18.63 (15.58–22.21)	−2.39 (−3.20–1.57)	24.42 (20.62–29.11)	0.21 (0.18–0.25)	−2.92 (−3.93–1.90)
	Caribbean	1534.14 (1161.42–1998.48)	8.35 (6.31–10.90)	−0.86 (−0.92–0.79)	17594.46 (15909.11–19387.03)	96.06 (86.83–105.86)	−0.62 (−0.64–0.60)	39664.79 (30209.95–52468.76)	216.07 (164.50–286.03)	−0.80 (−1.13–0.47)	650.67 (490.95–866.90)	3.54 (2.67–4.72)	−0.83 (−1.17–0.49)
	Central Asia	3664.08 (2803.05–4755.23)	9.31 (7.06–12.19)	−1.39 (−1.52–1.26)	33888.06 (30401.03–37603.19)	86.77 (77.63–96.51)	−1.04 (−1.08–0.99)	55040.55 (47783.92–62549.14)	139.68 (121.21–158.84)	−2.37 (−3.18–1.56)	850.29 (733.04–972.47)	2.14 (1.85–2.45)	−2.48 (−3.32–1.62)
	Central Europe	1583.98 (1055.36–2278.45)	4.15 (2.70–6.12)	−2.49 (−2.55–2.42)	25327.07 (22134.21–28809.65)	67.97 (59.18–77.48)	−1.29 (−1.34–1.24)	27292.07 (24618.20–30160.94)	68.24 (61.33–75.60)	−3.38 (−3.90–2.86)	401.02 (362.78–438.82)	0.97 (0.88–1.06)	−3.79 (−4.33–3.25)
	Central Latin America	4517.60 (2960.57–6623.37)	4.47 (2.93–6.55)	−1.87 (−1.91–1.83)	85437.40 (74093.23–97819.71)	84.51 (73.29–96.76)	−1.19 (−1.22–1.15)	82732.41 (72688.27–93451.07)	81.94 (71.99–92.56)	−2.16 (−2.59–1.73)	1285.65 (1122.91–1460.32)	1.27 (1.11–1.45)	−2.22 (−2.68–1.76)
	Central Sub-Saharan Africa	4990.16 (3677.89–6696.74)	10.15 (7.53–13.53)	−1.19 (−1.23–1.15)	48621.13 (43793.95–53775.37)	96.86 (87.65–106.65)	−1.04 (−1.07–1.00)	98903.10 (66224.46–140863.59)	200.06 (134.09–284.55)	−1.27 (−1.31–1.23)	1547.94 (1009.79–2247.73)	3.20 (2.09–4.63)	−1.26 (−1.30–1.21)
	East Asia	51831.73 (35427.46–72444.08)	9.57 (6.47–13.53)	−1.32 (−1.40–1.25)	587048.25 (490872.47–701406.38)	111.55 (92.98–133.45)	−1.09 (−1.14–1.05)	1161835.99 (975269.74–1364883.67)	211.92 (177.62–249.33)	−1.23 (−1.58–0.88)	18472.87 (15249.21–21945.53)	3.30 (2.72–3.93)	−1.23 (−1.65–0.81)
	Eastern Europe	6327.32 (4385.31–8783.50)	7.76 (5.30–10.94)	−0.15 (−0.36–0.06)	48132.70 (39727.57–57884.76)	63.16 (51.91–76.12)	−0.31 (−0.36–0.26)	119059.30 (106967.94–130034.08)	142.85 (128.12–156.16)	−0.08 (−1.09–0.94)	1986.99 (1776.29–2178.86)	2.32 (2.07–2.55)	0.03 (−1.04–1.11)
	Eastern Sub-Saharan Africa	16866.04 (12389.62–22369.48)	10.69 (7.90–14.06)	−2.01 (−2.06–1.96)	164610.49 (145972.33–184179.14)	102.45 (91.23–114.35)	−1.29 (−1.32–1.27)	425456.28 (331766.66–522361.16)	265.80 (207.9–326.11)	−1.72 (−1.81–1.63)	6684.80 (5164.22–8269.91)	4.27 (3.31–5.28)	−1.73 (−1.82–1.65)
	High-income Asia Pacific	2686.64 (1612.60–4219.94)	4.98 (2.93–7.96)	−2.33 (−2.50–2.15)	51304.73 (44328.98–58967.57)	96.21 (82.86–110.85)	−1.34 (−1.40–1.28)	30568.08 (26932.45–34766.53)	54.47 (47.75–62.19)	−3.21 (−3.55–2.87)	394.65 (359.27–443.98)	0.68 (0.62–0.77)	−3.67 (−4.21–3.12)
	High-income North America	3984.32 (2295.02–6279.85)	3.15 (1.79–5.00)	−0.76 (−0.95–0.56)	98476.78 (80545.51–119223.48)	78.08 (63.74–94.67)	−0.33 (−0.43–0.24)	59209.37 (53590.99–65916.91)	45.88 (41.45–51.17)	−0.93 (−1.33–0.53)	790.93 (729.68–846.23)	0.60 (0.56–0.65)	−1.12 (−1.67–0.56)
	North Africa and Middle East	18161.06 (13133.26–24486.78)	7.01 (5.05–9.48)	−1.81 (−1.89–1.72)	251640.11 (227355.46–277565.32)	97.36 (87.88–107.47)	−1.22 (−1.25–1.20)	454447.06 (378706.20–544484.17)	175.63 (146.29–210.39)	−2.36 (−2.52–2.20)	7012.84 (5745.63–8486.67)	2.69 (2.20–3.26)	−2.47 (−2.63–2.31)
	Oceania	550.72 (413.75–727.84)	10.24 (7.73–13.47)	−0.94 (−0.98–0.91)	5616.70 (5050.70–6196.08)	102.89 (92.71–113.32)	−0.65 (−0.72–0.59)	23540.72 (16053.43–33137.04)	436.97 (298.37–614.27)	−0.65 (−0.77–0.53)	384.20 (256.77–547.37)	7.19 (4.81–10.22)	−0.66 (−0.78–0.54)
	South Asia	60390.59 (39902.90–86769.88)	7.80 (5.16–11.2)	−0.88 (−0.96–0.80)	603502.34 (499908.83–720516.71)	77.55 (64.28–92.57)	−0.56 (−0.60–0.53)	1106005.25 (886842.71–1334585.56)	142.95 (114.69–172.45)	−1.35 (−1.76–0.93)	17527.77 (13850.84–21454.02)	2.27 (1.80–2.78)	−1.39 (−1.84–0.94)
	Southeast Asia	38204.61 (27835.16–51232.82)	13.49 (9.81–18.11)	−1.01 (−1.04–0.97)	362417.29 (314696.29–416860.48)	128.46 (111.49–147.80)	−0.72 (−0.74–0.70)	1060304.71 (919909.88–1243743.98)	374.15 (324.55–438.84)	−1.08 (−1.17–0.99)	17191.06 (14754.73–20353.19)	6.05 (5.19–7.16)	−1.10 (−1.22–0.99)
	Southern Latin America	1743.55 (1158.54–2517.15)	6.67 (4.41–9.67)	−2.37 (−2.45–2.29)	29648.61 (26873.37–32471.17)	112.91 (102.27–123.76)	−1.33 (−1.38–1.28)	21221.92 (18689.66–24046.03)	80.41 (70.81–91.13)	−3.60 (−4.41–2.78)	292.10 (255.64–329.94)	1.10 (0.96–1.24)	−4.00 (−4.84–3.15)
	Southern Sub-Saharan Africa	2494.99 (1738.20–3462.18)	7.22 (5.02–10.03)	−2.41 (−2.56–2.27)	24406.29 (20405.60–29086.84)	70.80 (59.17–84.39)	−1.45 (−1.52–1.38)	76043.05 (64862.58–89571.93)	220.13 (187.66–259.39)	−1.89 (−2.89–0.88)	1247.92 (1060.99–1478.86)	3.60 (3.06–4.27)	−1.95 (−3.03–0.85)
	Tropical Latin America	4578.26 (2939.74–6663.71)	4.95 (3.16–7.24)	−3.53 (−3.59–3.46)	58906.34 (48659.51–70585.22)	64.4 (53.10–77.27)	−1.80 (−1.84–1.75)	99584.15 (93650.47–105543.08)	106.86 (100.45–113.27)	−3.57 (−3.98–3.16)	1636.68 (1541.36–1736.86)	1.74 (1.64–1.85)	−3.70 (−4.12–3.29)
	Western Europe	3506.21 (1917.30–5838.32)	2.61 (1.40–4.40)	−2.09 (−2.15–2.02)	73781.60 (61987.24–86624.36)	54.67 (45.79–64.26)	−1.06 (−1.09–1.03)	35450.90 (31856.61–39491.53)	25.58 (22.92–28.57)	−4.09 (−4.73–3.44)	446.48 (421.19–473.26)	0.32 (0.30–0.33)	−4.72 (−5.48–3.96)
	Western Sub-Saharan Africa	17506.33 (12507.59–23914.44)	10.17 (7.34–13.75)	−1.01 (−1.07–0.95)	255538.31 (229358.40–283784.16)	142.43 (128.22–157.89)	−0.70 (−0.72–0.68)	372439.39 (286188.49–454279.66)	215.14 (166.00–262.04)	−1.27 (−1.35–1.18)	5672.28 (4261.30–6982.02)	3.36 (2.53–4.12)	−1.32 (−1.41–1.23)

The metrics include incidence, prevalence, disability-adjusted life years (DALYs), and deaths. Data are presented as absolute numbers for 2021 (Num_2021) and age-standardized rates (ASR_2021), with 95% uncertainty intervals (UI) in parentheses. The Average Annual Percentage Change (AAPC) reflects trends from 1990 to 2021, with 95% confidence intervals (CI) provided. ASR denotes the rate per 100,000 population, standardized to the 2010 global standard population. The data are stratified by global, sex, Sociodemographic Index (SDI) levels, and regions.

AYAs, Adolescents and Young Adults, defined as individuals aged 15–39 years; ICH, Intracerebral Hemorrhage, specifically spontaneous intracerebral hemorrhage; DALY, Disability-Adjusted Life Year; ASR, Age-Standardized Rate, typically expressed as the incidence or prevalence per 100,000 population after adjusting for age differences; AAPC, Average Annual Percentage Change, indicating trends in rates over the specified period; UI, Uncertainty Interval, representing the range of uncertainty around the estimate; CI, Confidence Interval, denoting the range in which the true value is expected to fall with 95% confidence; SDI, Sociodemographic Index, a composite indicator of a region's socioeconomic development level.

In 2021, the highest number of incident cases, prevalence, deaths, and DALYs among AYAs globally were observed in regions with middle-level SDI, whereas the lowest values for these indicators were recorded in High SDI regions. Similarly, the highest age-standardized rates for the number of incident cases, prevalence, deaths, and DALYs were found in Low SDI regions, with the lowest rates occurring in High SDI regions. Compared to 1990, the number of incident cases in 2021 increased in low-middle SDI regions, whereas it decreased in high, high-middle, middle, and low SDI regions. The number of prevalent cases, deaths, and DALYs showed a similar trend, with increases in Low-middle and Low SDI regions, and decreases in High, High-middle, and Middle SDI regions. Globally, the ASIR, ASPR, ASDR, and ASMR all decreased across countries with varying levels of SDI (Table 1 and Supplementary Table 1).

For the 21 regions considered, the 2021 ICH indicators demonstrated significant regional disparities. Specifically, Australasia demonstrated the lowest ASIR, ASPR, ASMR, and ASDR. In contrast, Southeast Asia had the highest ASIR, Western Sub-Saharan Africa had the highest ASPR, and Oceania had the highest ASMR and ASDR. In terms of actual numbers, South Asia had the distinction of having the highest number of incident cases and prevalent cases, while East Asia recorded the highest number of deaths and DALYs. These indicators' lowest values were similarly observed in Australasia (Table 1 and Supplementary Table 1).

For the 204 countries considered, the 2021 ICH indicators demonstrated significant intercountry variations. Specifically, the highest ASIR and ASPR were observed in Kiribati, while the lowest ASIR was seen in Switzerland and the lowest ASPR in Lithuania. The highest ASMR and ASDR were both noted in Nauru, with the lowest values for these indicators observed in Norway. In terms of absolute numbers, the highest number of incident cases and prevalent cases occurred in China, with the lowest numbers recorded in Tokelau. Similarly, the highest number of deaths and DALYs were also observed in China, while the lowest numbers were observed in San Marino (Supplementary Table 2).

### 3.2 Joinpoint analysis

Through joinpoint analysis, globally among AYAs, the incidence, prevalence, mortality, and DALYs associated with ICH were analyzed and showed overall decreasing trends (AAPC: −1.24; AAPC: −0.9; AAPC: −1.4; AAPC: −1.37).

The incidence of ICH showed a slow decrease from 1990 to 1997 (APC: −0.32; 95% CI: −0.43 to −0.21), followed by a faster decline from 1997 to 2006 (APC: −1.05; 95% CI: −1.14 to −0.96). From 2006 to 2014, the incidence decreased at an even faster rate (APC: −2.91; 95% CI: 3.02 to 2.81). From 2014 to 2019, the incidence had a slight increase (APC: 0.04; 95% CI: −0.21 to 0.30). From 2019 to 2021, the incidence began to decrease again (APC: −1.68; 95% CI: −2.48 to −0.88). Over the entire period from 2006 to 2019, the AAPC *p*-value did not show a statistically significant difference.

The prevalence of ICH declined only between 1990 and 2019, with the fastest decrease occurring between 2005 and 2015 (APC: −1.61; 95% CI: −1.65 to −1.58). From 2015 to 2019, the decrease in prevalence slowed (APC: −0.38; 95% CI: −0.58 to −0.19). After 2019, the prevalence started to increase (APC: 1.34; 95% CI: 0.95 to 1.73).

The mortality rate for ICH decreased overall from 1990 to 2021, with the fastest decline occurring between 2009 and 2012 (AAPC: −3.12; 95% CI: −4.40 to −1.81).

**Figure 1:**
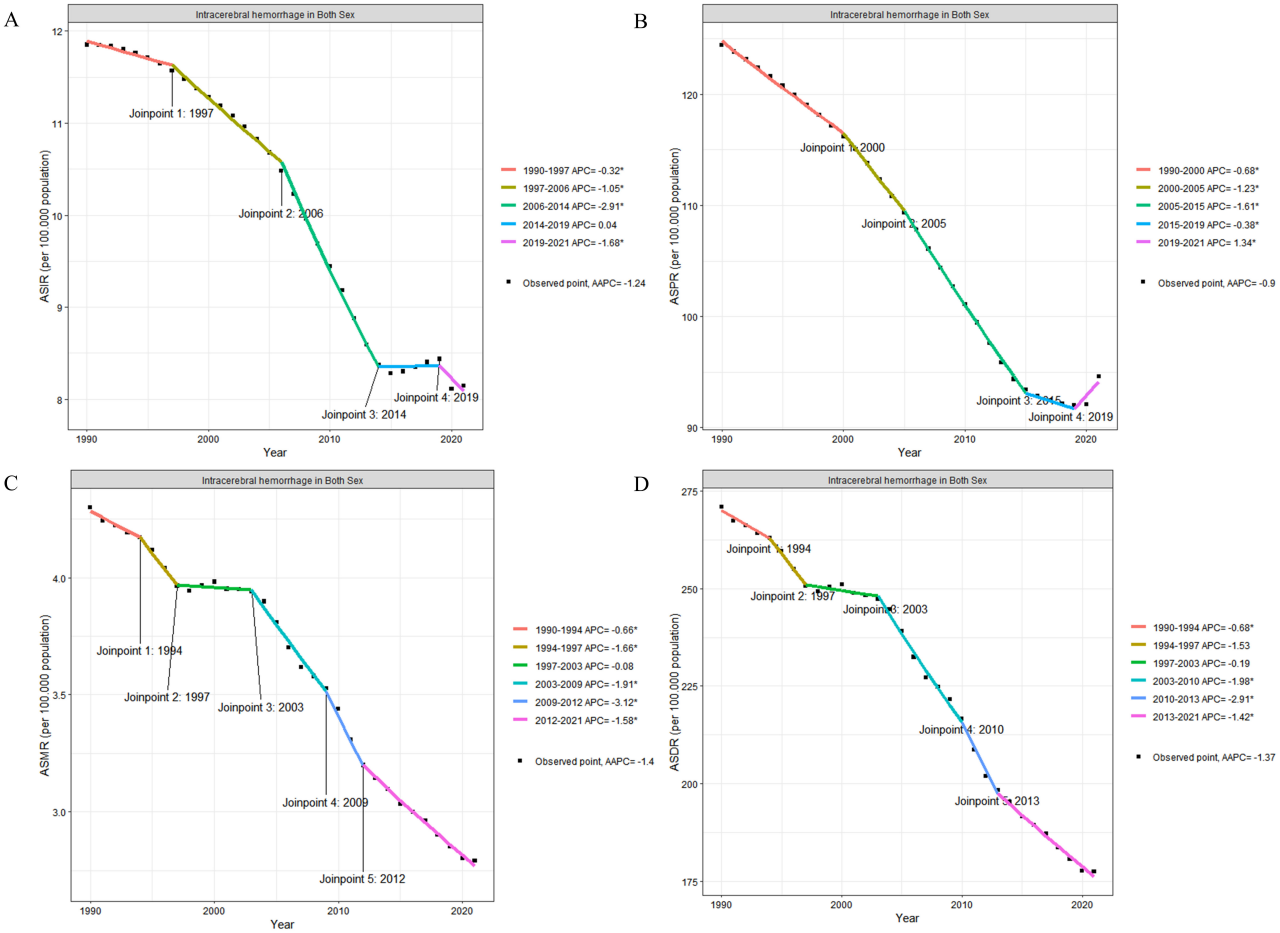
Temporal trends in age-standardized intracerebral hemorrhage rates in adolescents and young adults (both sexes) from 1990 to 2021. **(A)** ASIR of ICH per 100,000 population. The trend shows significant changes at four joinpoints: 1997, 2006, 2014, and 2019. The APC for each segment is as follows: 1990–1997 APC = −0.32*, 1997–2006 APC = −1.05*, 2006–2014 APC = −2.91*, 2014–2019 APC = 0.04, and 2019–2021 APC = −1.68*. The AAPC over the entire period is −1.24*. **(B)** ASPR of ICH per 100,000 population. Five joinpoints were identified: 2000, 2005, 2014, and 2019, with corresponding APCs: 1990–2000 APC = −0.68*, 2000–2005 APC = −1.23*, 2005–2015 APC = −1.61*, 2015–2019 APC = −0.38*, and 2019–2021 APC = 1.34*. The AAPC over the entire period is −0.9*. **(C)** ASMR of ICH per 100,000 population. Significant changes in the trend were observed at five joinpoints: 1994, 1997, 2003, 2009, and 2012. The APCs for each segment are: 1990–1994 APC = −0.66*, 1994–1997 APC = −1.68*, 1997–2003 APC = −0.19, 2003–2009 APC = −4.91*, 2009–2012 APC = −3.12*, and 2012–2021 APC = −1.58*. The overall AAPC is −1.4*. **(D)** ASDR of ICH per 100,000 population. Four joinpoints were identified: 1994, 1997, 2003, and 2013. The APCs are: 1990–1994 APC = −0.68*, 1994–1997 APC = −1.53*, 1997–2003 APC = −0.19, 2003–2010 APC = −1.98*, 2010–2013 APC = −2.91*, and 2013–2021 APC = −1.42*. The overall AAPC is −1.37*. APC, Annual Percentage Change; AAPC, Average Annual Percentage Change; ASIR, Age-Standardized Incidence Rate; ASPR, Age-Standardized Prevalence Rate; ASMR, Age-Standardized Mortality Rate; ASDR, Age-Standardized Disability-Adjusted Life Years; CI, Confidence Interval. Asterisks (*) indicate statistical significance at *p* < 0.05.

Regarding DALYs, from 1990 to 2021, the DALYs associated with ICH in AYAs populations showed an overall decreasing trend, but the rate of decrease varied across different periods. The fastest decrease occurred between 2010 and 2013 (APC: −2.91; 95% CI: −4.40 to −1.40). After 2013, the decrease in DALYs slowed down (APC: −1.42; 95% CI: −1.58 to −1.25; Figure 1, Supplementary Figure 1 and Supplementary Table 3).

### 3.3 Correlation with social development index

**Figure 2:**
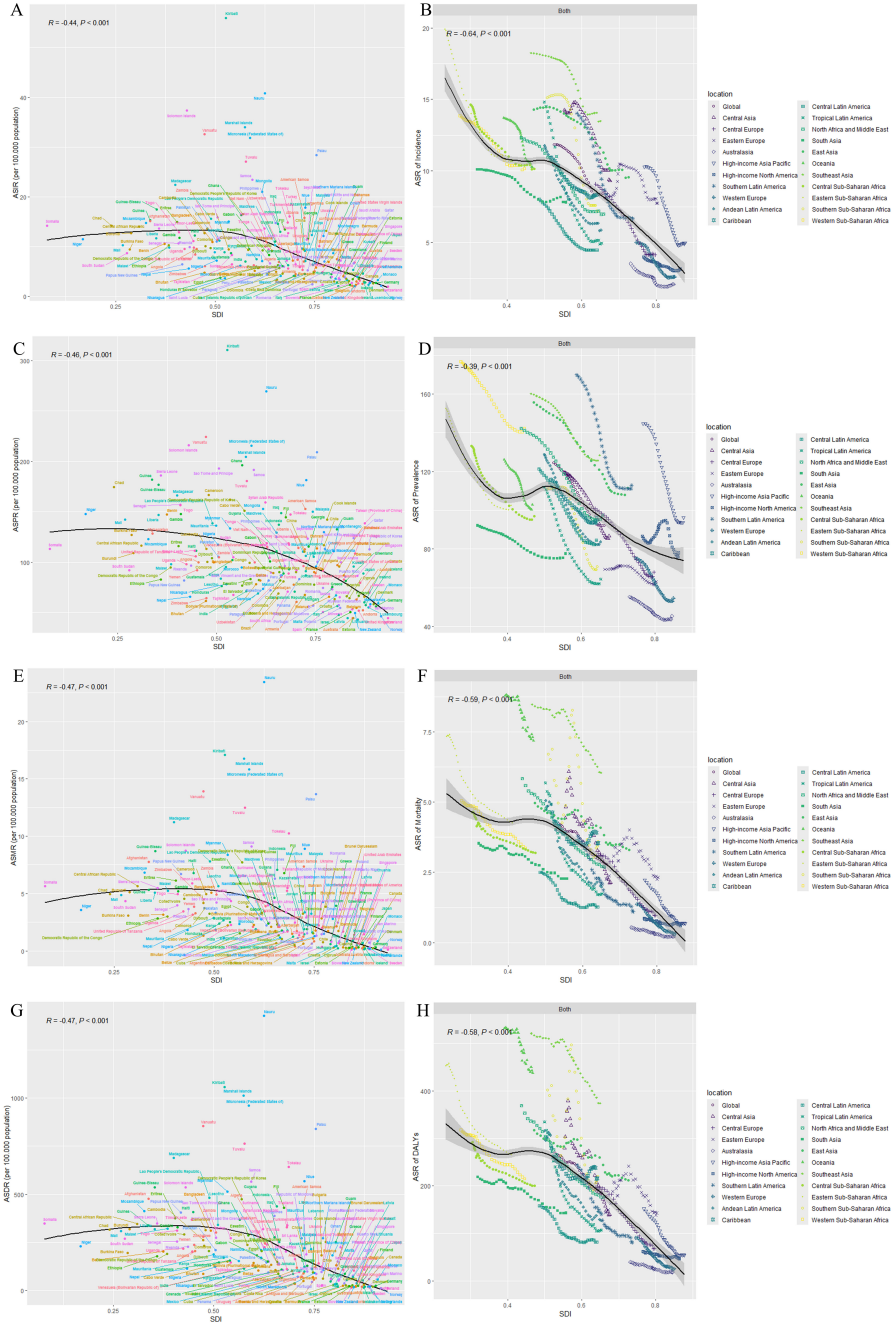
Correlation between SDI and age-standardized rates of intracerebral hemorrhage in adolescents and young adults (both sexes) from 1990 to 2021. **(A)** ASIR vs. SDI. The scatterplot shows the negative correlation between SDI and ASIR of ICH across 204 countries from 1990 to 2021 (*R* = −0.14, *P* < 0.001). Countries with higher SDI generally exhibit lower ASIRs. **(B)** Smoothed spline curve showing the relationship between SDI and ASIR across 21 global regions. As SDI increases, ASIR decreases, indicating a significant negative correlation (*R* = −0.64, *P* < 0.001). **(C)** ASPR vs. SDI. A negative correlation is observed between SDI and ASPR (*R* = −0.15, *P* < 0.001), implying that higher SDI countries have lower prevalence rates. **(D)** Smoothed spline curve showing the relationship between SDI and ASPR across 21 global regions. The negative correlation is evident with *R* = −0.39 (*P* < 0.001). **(E)** ASMR vs. SDI. The scatterplot indicates a significant negative correlation (*R* = −0.17, *P* < 0.001), showing that as SDI increases, mortality rates decrease. **(F)** Smoothed spline curve showing the relationship between SDI and ASMR across regions, confirming the inverse relationship between SDI and mortality rates (*R* = −0.59, *P* < 0.001). **(G)** ASDR vs. SDI. A clear negative correlation is observed between SDI and ASDR (*R* = −0.17, *P* < 0.001), with higher SDI countries exhibiting lower disease burden. **(H)** Smoothed spline curve showing the relationship between SDI and ASDR across global regions, further supporting the negative correlation (*R* = −0.58, *P* < 0.001). ASIR, Age-Standardized Incidence Rate; ASPR, Age-Standardized Prevalence Rate; ASMR, Age-Standardized Mortality Rate; ASDR, Age-Standardized Disability-Adjusted Life Years; SDI, Social Development Index; R, Pearson correlation coefficient; P, *p*-value. Tools and software: figures were generated using R software (version 4.2.2) with the ggplot2 package. Spline curves were fitted using the geom_smooth() function to visualize trends across global regions. Data points represent individual countries, and regional groupings are color-coded. Statistical tests: Pearson's correlation coefficient (R) was calculated to assess the strength of the relationship between SDI and each health indicator. A *p*-value < 0.001 was considered statistically significant.

The analysis of data from 1990 to 2021 across 204 countries demonstrates a significant negative correlation between ASIR, ASPR, ASMR, and ASDR with SDI. As SDI increases, these health burden indicators show a clear downward trend across countries. At the regional level, among the 21 global regions, the age-standardized incidence rates in Central Asia and Southeast Asia are higher than expected based on their respective SDI levels. Additionally, in High-income Asia Pacific, Southern Latin America, Southeast Asia, and Western Sub-Saharan Africa, the age-standardized prevalence rates are significantly higher than anticipated given their SDI. Furthermore, Oceania and Southeast Asia exhibit ASMR and DALYs that are notably higher than expected based on their SDI (Figure 2).

### 3.4 Health inequality analysis

**Figure 3:**
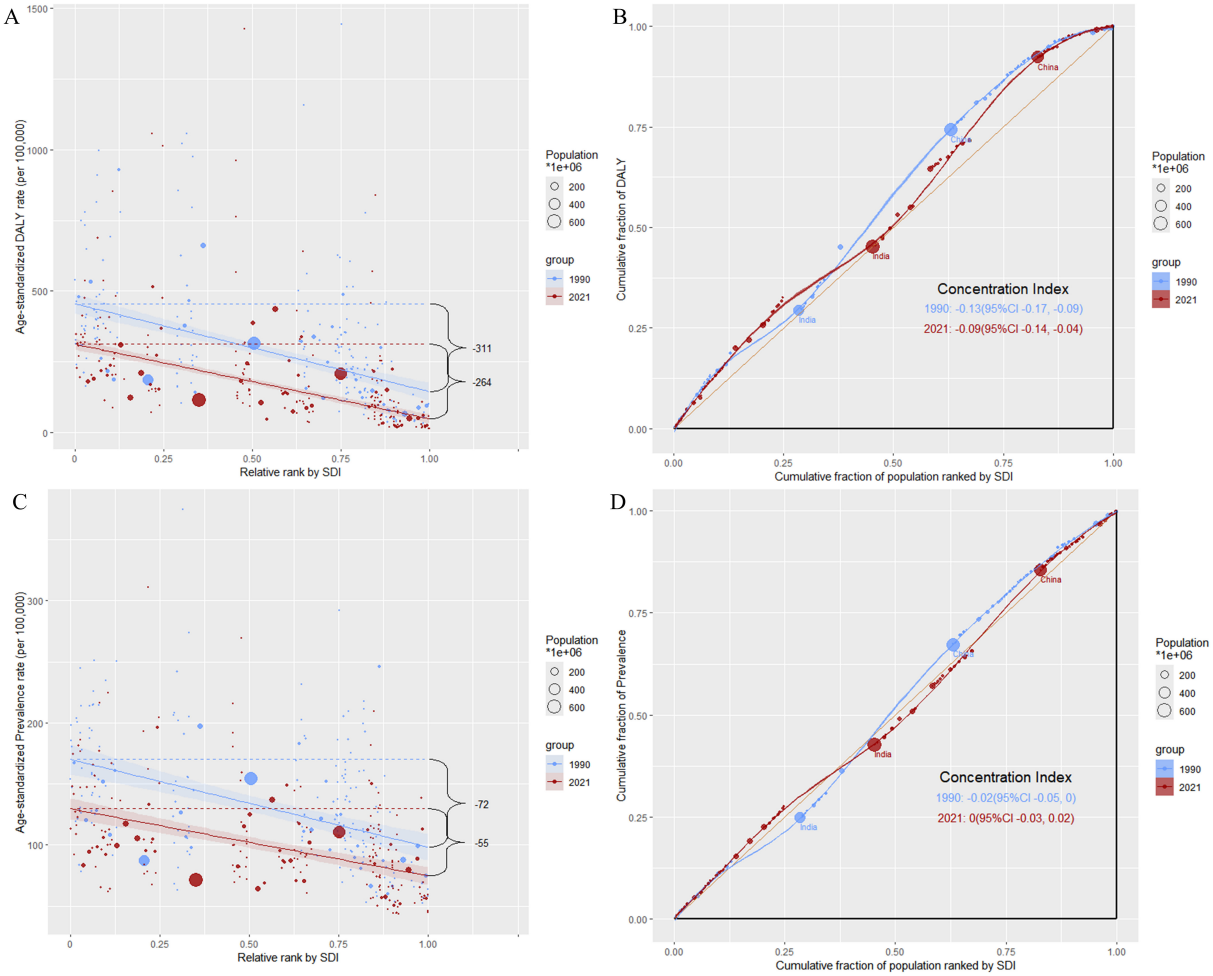
Analysis of social inequality in intracerebral hemorrhage burden among adolescents and young adults in 1990 and 2021. **(A)** Age-standardized DALY rate vs. relative rank by SDI. The scatterplot shows the age-standardized DALY rate per 100,000 population about the SDI rank for 204 countries in 1990 and 2021. The SII indicates a decrease in inequality, with the gap narrowing from 311 per 100,000 in 1990 to 264 per 100,000 in 2021. Bubble size represents population size, with larger bubbles indicating more populous countries. **(B)** Concentration curve for DALY rates. The concentration curve illustrates inequality in DALY distribution across countries ranked by SDI. The CI improved from −0.13 (95% CI: −0.17, −0.09) in 1990 to −0.09 (95% CI: −0.14, −0.04) in 2021, indicating a reduction in inequality, where negative values reflect a disproportionate burden on lower-SDI countries. **(C)** Age-standardized prevalence rate vs. relative rank by SDI. The scatterplot shows the age-standardized prevalence rate of ICH per 100,000 population relative to SDI rank for 204 countries in 1990 and 2021. The SII shows a reduction in prevalence inequality, with the gap narrowing from 72 per 100,000 in 1990 to 55 per 100,000 in 2021. **(D)** Concentration curve for prevalence rates. The concentration curve for prevalence rates exhibits a slight shift toward equity from 1990 to 2021. The concentration index (CI) improved from −0.02 (95% CI: −0.05, 0.00) in 1990 to 0.00 (95% CI: −0.03, 0.02) in 2021, indicating less pronounced inequality in prevalence distribution by SDI. SDI, Social Development Index; DALY, Disability-Adjusted Life Years; SII, Slope Index of Inequality; CI, Concentration Index.

This study reveals a persistent narrowing trend in the gap of DALYs caused by ICH among AYAs globally from 1990 to 2021.Despite the ongoing substantial burden of health inequality in regions with low SDI. The slope index of inequality in health shows a significant gap in DALYs rates between countries with the lowest and highest SDI narrowed from 310.9 (95% CI: 370.7 to 251.2) per 100,000 people in 1990 to 263.8 (95% CI: 228.0 to 299.7) per 100,000 people in 2021, reflecting a reduction in global health inequalities. The concentration index for assessing health inequality decreased from −0.13 (95% CI: −0.17 to −0.09) in 1990 to −0.09 (95% CI: −0.14 to −0.04) in 2021, indicating that ICH still disproportionately affects populations with lower socioeconomic status within the AYAs group. The analysis of health inequality for prevalence also indicates a similar trend. The slope index of inequality shows that the gap in ASPR between countries with the lowest and highest SDI narrowed from 71.8 (95% CI: 53.6 to 90.0) per 100,000 people in 1990 to 54.9 (95% CI: 41.5 to 68.4) per 100,000 people in 2021. The concentration index for assessing health inequality decreased from −0.02 (95% CI: −0.05 to 0) in 1990 to 0 (95% CI: −0.03 to 0.02) in 2021 (Figure 3).

### 3.5 Bayesian age-period-cohort prediction model

By 2040, the number of global ICH cases is expected to reach 4,142,187, with 2,135,210 cases in males and 1,978,532 cases in females, representing a growth rate of 31.1% compared to 2021. Projections indicate that the number of new cases will reach 249,137, with 149,988 cases in males and 100,373 cases in females, showing a growth rate of 0.9% compared to 2021. The number of deaths is expected to be 72,665, with 46,783 cases in males and 26,144 cases in females, indicating a decline rate of 14.5% compared to 2021. The ASDR is projected to be 4768727.8, with 2966180.1 in males and 1826859.4 in females, showing a decline rate of 11.4% compared to 2021.

**Figure 4:**
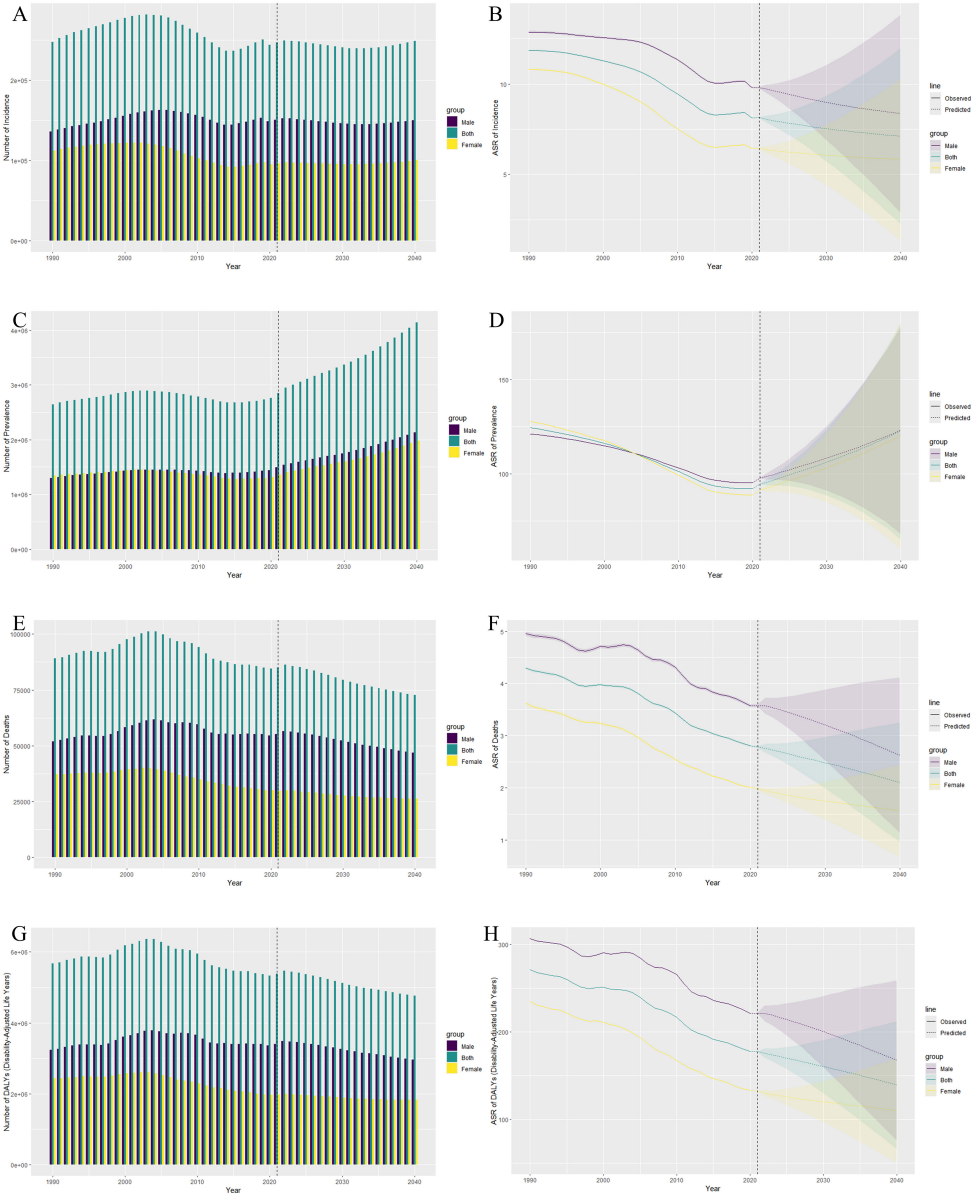
Observed and predicted trends of intracerebral hemorrhage (ICH) burden among adolescents and young adults from 1990 to 2040 by sex. **(A)** The bar plot shows the observed number of ICH incidence cases (1990–2021) and the predicted number (2022–2040) for both sexes, males, and females. The predicted increase in ICH cases is more pronounced in males. **(B)** The line graph shows the observed ASIR for both sexes, males, and females from 1990 to 2021, with predicted trends from 2022 to 2040. The ASIR is expected to increase slightly in males and stabilize in females. **(C)** The bar plot shows the observed and predicted number of prevalent cases, highlighting a gradual increase in prevalence, especially in males, over the forecast period. **(D)** The ASPR for both sexes, males, and females shows a slight increase in males and a more stable trend in females during the prediction period. **(E)** The bar plot illustrates the number of deaths due to ICH, with a predicted decrease in both sexes, but males continue to have a higher burden. **(F)** The line graph demonstrates a declining trend in ASMR from 1990 to 2040, with males experiencing a steeper decline compared to females. **(G)** The bar plot shows the projected decline in the number of DALYs lost, with males consistently bearing a higher burden than females. **(H)** The ASDR shows a declining trend for both sexes, with a steeper decline in males during the prediction period. ASIR, Age-Standardized Incidence Rate; ASPR, Age-Standardized Prevalence Rate; ASMR, Age-Standardized Mortality Rate; ASDR, Age-Standardized Disability-Adjusted Life Years Rate; DALY, Disability-Adjusted Life Years.

According to the forecast data, from 2021 to 2040, the ASDR and ASMR due to ICH are expected to continue to decline in both males and females, with males declining at a faster rate than females. However, during the same period, the ASIR and ASPR of ICH in both males and females are expected to continue to increase, with males having higher numbers than females (Figure 4 and Supplementary Table 4).

## 4 Discussion

From 1990 to 2021, the global burden of intracerebral hemorrhage in adolescents and young adults, aged 15–39 years, demonstrated a significant downward trend. This reduction is primarily reflected in the decreases in age-standardized rate of incidence, prevalence, mortality, and disability-adjusted life years. These trends highlight the considerable advancements made globally in healthcare infrastructure, chronic disease management, and the treatment of acute ICH cases (14). However, compared to other age groups, the ICH burden in AYAs populations is uniquely influenced by lifestyle factors, social roles, and health behaviors, resulting in distinct trends (15).

In terms of gender differences, the burden of ICH has consistently been higher in males than in females, a trend that is particularly pronounced within the AYAs group. Males are more frequently engaged in high-risk occupations, such as construction, transportation, and manufacturing (16). These jobs often involve high physical exertion and exposure to hazardous environmental conditions which substantially elevate the risk of ICH (17). Additionally, males in this age group tend to engage in unhealthy lifestyle behaviors, including higher rates of smoking, alcohol consumption, and dietary patterns characterized by high salt and fat intake (18). These behaviors are well-established risk factors for ICH. Furthermore, insufficient health awareness exacerbates the problem, as many males neglect regular health screenings, leading to unmanaged hypertension and other chronic conditions, which further increase the risk of ICH (18). However, while the overall ICH burden is higher in males, specific risk factors in females may contribute to comparable prevalence rates in the AYA population (19). Moyamoya disease, more common in females, is a significant cause of ICH, particularly in East Asian populations (20). Additionally, hypertensive disorders of pregnancy, such as preeclampsia and eclampsia, increase ICH risk during the perinatal period (21). These conditions can lead to acute elevations in blood pressure, increasing the likelihood of hemorrhagic stroke. Given these considerations, while male-dominated occupational hazards and unhealthy lifestyle behaviors may explain the higher overall ICH burden in males, the influence of female-specific factors, including vascular abnormalities and pregnancy-related hypertensive complications, should not be overlooked. Future studies should further investigate these gender-specific risk factors to inform targeted prevention strategies.

In terms of regional disparities, high-income countries, benefiting from improvements in the Social Development Index, have achieved significant control over the ICH burden (22). AYAs populations in these nations have greater access to healthcare resources, including hypertension screening, emergency medical services, and advanced medical technologies (23). Long-term policy interventions, optimization of healthcare resources, and heightened health awareness in these regions have contributed to the substantial decline in ICH incidence and mortality among AYAs populations. However, in low- and middle-income countries, the AYAs population continues to face a higher ICH burden, with slower rates of improvement. Factors such as weak healthcare infrastructure, inadequate medical facilities, and a lack of health education hinder access to preventive health services—particularly in the early management of hypertension and other chronic diseases. This not only leads to higher ICH incidence but also exacerbates health disparities between urban and rural populations and between wealthier and poorer socioeconomic groups (24).

The vulnerability of AYAs populations in the context of socioeconomic inequalities further exacerbates their health burden. In low-income countries, economic disparities limit the ability of AYAs to access regular health check-ups and preventive services, especially in rural and remote areas (25). Economic pressures lead many young individuals to neglect their health, resulting in poorly managed risk factors like hypertension, which ultimately increases the risk of ICH (26). Additionally, the unequal distribution of healthcare resources in low-income countries means that impoverished AYAs populations often struggle to obtain adequate healthcare services. This unequal distribution of resources significantly heightens the health burden for AYAs beyond what would be expected (26). In some low-income settings, traditional beliefs also contribute to this disparity, as youth are often perceived as inherently healthy, leading to negligence in the prevention of chronic diseases. This mindset further exacerbates health inequalities (27).

Temporal trend analyses show that the global ICH burden in AYAs populations underwent significant changes between 1990 and 2021, particularly from 2006 to 2014 when the decline in incidence and mortality rates accelerated. This improvement is closely linked to the effectiveness of global chronic disease management policies, such as hypertension screening, smoking cessation, alcohol reduction, and the promotion of healthy diets (28, 29). However, since 2014, certain regions have experienced a rebound in ICH incidence, particularly within the AYAs population. This rebound may be attributed to insufficient policy coverage and changes in lifestyle behaviors; the increasing prevalence of obesity, diabetes, and other lifestyle-related chronic conditions among AYAs is likely contributing to the rising ICH burden (30–32). Moreover, AYAs in modern societies face substantial work-related and life stress, especially in low- and middle-income countries (33). Economic pressures and unstable working conditions drive many to cope through harmful behaviors such as smoking and excessive drinking, further increasing their risk of ICH (34).

Between 2019 and 2021, the prevalence of ICH increased over time, whereas the incidence exhibited a declining trend, a pattern consistently observed across all SDI regions. One possible explanation for these trends is the impact of the COVID-19 pandemic, which disrupted healthcare delivery, altered patient behavior, and affected disease reporting (35). Reports from multiple countries suggest that the reallocation of medical resources to COVID-19 care overwhelmed healthcare systems, delaying diagnoses for non-emergency conditions like ICH and likely contributing to the decline in incidence, reflecting underestimation rather than a true reduction in cases (36). Additionally, fear of infection led many patients to avoid hospitals, resulting in undiagnosed mild-to-moderate ICH cases (37). Simultaneously, disruptions in hospitalization and rehabilitation services delayed admissions and follow-up care, increasing the number of surviving ICH patients with prolonged disease duration, which may explain the rise in prevalence despite the decline in incidence (38). These trends underscore the broader impact of the pandemic on healthcare access and patient management, highlighting the need for future studies to address underreporting and assess long-term epidemiological shifts as healthcare systems recover.

Looking ahead, Bayesian Age-Period-Cohort models predict that the global AYAs ICH burden may continue to rise as chronic diseases proliferate. Although future mortality rates and disability-adjusted life years are expected to continue declining, incidence and prevalence may increase, particularly among males. Lifestyle risk factors—such as obesity, smoking, and alcohol consumption—will likely remain the primary drivers of this burden. As globalization and urbanization progress, the growing pressures of daily living and work may further deteriorate health behaviors in AYAs populations. This not only raises the risk of chronic diseases but could also lead to a resurgence of ICH incidence (39). Furthermore, many low-income countries lack tailored chronic disease management systems for AYAs, leaving this population vulnerable to unmanaged health risks. In the future, this systemic lag in healthcare could exacerbate the impact of chronic disease epidemics on the AYAs population (40).

While this study provides a thorough examination of the global ICH burden in AYAs using GBD 2021 data, it does have limitations. First, the quality and availability of data from low-income countries are suboptimal or lacking, potentially underestimating the actual burden. Second, the study does not fully explore rural-urban and socioeconomic disparities within countries, nor does it adequately account for the influence of cultural factors on health behaviors (41). Lastly, the predictive models do not fully consider potential future policy changes, advancements in medical technology, or the impact of public health crises, such as the COVID-19 pandemic (42). Future research should endeavor to improve data quality, refine the analysis of internal disparities, and incorporate more dynamic social and cultural factors.

In conclusion, the global ICH burden among AYAs populations shows significant regional and gender differences, influenced by socioeconomic factors, lifestyle behaviors, and healthcare systems. Public health policies moving forward should prioritize the specific health needs of AYAs populations, optimize resource allocation, enhance health education, and focus on chronic disease prevention and management to mitigate the long-term impacts of ICH and other major diseases on this critical age group.

## Data Availability

The original contributions presented in the study are included in the article/Supplementary material, further inquiries can be directed to the corresponding authors.
