# Supplementary material for: Global, regional, and national burden and trends of intracerebral hemorrhage among adolescents and young adults aged 15–39 years from 1990 to 2021: a comprehensive trend analysis based on the global burden of disease study 2021
Source: Front Neurol. 2025 Apr 23;16:1538413. doi: 10.3389/fneur.2025.1538413 (PMC12056743; doi:10.3389/fneur.2025.1538413)

**Table S1. Regional Trends in ICH Burden Among AYAs in 2021.**

|  |  | **Incidence** | | **Prevalence** | | **DALYs** | | **Deathes** | |
| --- | --- | --- | --- | --- | --- | --- | --- | --- | --- |
| **Characteristics** |  | **Num_1990 (95% UI)** | **ASR_1990 (95% UI)** | **Num_1990 (95% UI)** | **ASR_1990 (95% UI)** | **Num_1990 (95% UI)** | **ASR_1990 (95% UI)** | **Num_1990 (95% UI)** | **ASR_1990 (95% UI)** |
| Global | Global | 247927.77(166069.32-350827.75) | 11.85(7.96-16.69) | 2643929.28(2215751.01-3134225.18) | 124.44(104.42-147.40) | 5673698.79(5168690.3-6151452.09) | 271.00(246.98-293.66) | 89160.94(81016.40-96646.15) | 4.3(3.91-4.66) |
| Sex | Male | 135700.74(91175.18-192527.98) | 12.86(8.66-18.19) | 1299381.52(1096813.59-1534445.85) | 121.09(102.34-142.87) | 3236405.42(2771671.52-3692983.69) | 306.24(262.42-349.04) | 51896.62(44127.52-59489.63) | 4.95(4.22-5.67) |
|  | Female | 112227.04(73628.04-162830.80) | 10.81(7.11-15.61) | 1344547.76(1121724.31-1602633.2) | 127.87(106.83-152.28) | 2437293.38(2124316.67-2762331.49) | 234.81(204.89-265.85) | 37264.32(32196.71-42819.85) | 3.63(3.14-4.16) |
| Sociodemographic index | High SDI | 22538.6(14719.42-33305.92) | 6.30(4.09-9.36) | 349077.31(294712.17-409912.64) | 98.21(82.77-115.45) | 381827.33(353653.31-410491.06) | 106.03(98.15-114.11) | 5673.31(5323.39-6052.72) | 1.56(1.47-1.67) |
|  | High-middle SDI | 56362.72(37506.51-79834.31) | 12.54(8.35-17.75) | 604813.43(504976.28-722651.22) | 134.1(111.98-160.23) | 1178897.49(1052842.78-1315948.58) | 262.42(234.37-292.89) | 18400.25(16375.87-20689.34) | 4.1(3.65-4.61) |
|  | Middle SDI | 92351.36(60767.91-132919.13) | 13.28(8.78-18.98) | 971045.71(800326.74-1171364.78) | 135.81(112.18-163.66) | 2178996.89(1972194.26-2453537.66) | 314.11(284.38-353.45) | 34335.97(30977.78-38741.33) | 5.03(4.53-5.67) |
|  | Low-middle-SDI | 51738.17(35315.42-72406.76) | 12.53(8.60-17.39) | 484244.68(410164.66-569270.27) | 114.23(97.03-134.15) | 1353707.47(1144384.75-1563952.44) | 325.46(275.79-375.59) | 21610.72(18141.15-25031.01) | 5.28(4.44-6.11) |
|  | Low SDI | 24722.19(17452.48-33820.42) | 14.92(10.58-20.27) | 232521.13(202961.92-265103.92) | 136(119.01-154.98) | 574658.09(460016.26-676125.60) | 339.85(273.11-398.45) | 9050.65(7175.75-10728.31) | 5.47(4.35-6.46) |
| Regions | Andean Latin America | 1731.82(1289.28-2297.99) | 11.78（8.83-15.54） | 18717.9(16874.74-20614.97) | 128.79（116.42-141.53） | 46492.02(38248.70-56099.11) | 315.82（259.87-380.70） | 730.55(595.48-884.17) | 5.05（4.12-6.11） |
|  | Australasia | 291.89(176.15-442.32) | 3.52（2.12-5.36） | 4568.33(3755.21-5446.57) | 55.20（45.33-65.88） | 3308.64(2875.80-3793.01) | 39.80（34.59-45.63） | 45.2(38.9-52.41) | 0.54（0.47-0.63） |
|  | Caribbean | 1488.19（1109.07-1971.26） | 10.93（8.25-14.31） | 16371.59（14656.52-18153.74） | 115.75（103.98-128.01） | 38294.60（32033.40-45902.22） | 279.82（234.81-333.93） | 623.64（519.36-747.88） | 4.63（3.87-5.53） |
|  | Central Asia | 3842.68（2943.55-5003.54） | 14.26（11.01-18.48） | 32521.66（29015.68-36076.77） | 119.42（106.77-132.20） | 75423.33（69820.82-81266.67） | 279.61（258.89-301.15） | 1182.56（1096.39-1275.20） | 4.43（4.11-4.78） |
|  | Central Europe | 4504.60（3288.39-6034.01） | 9.08（6.57-12.28） | 48701.90（42349.31-55471.99） | 100.78（87.45-114.97） | 97823.88（92096.00-103687.22） | 196.40（184.72-208.34） | 1560.88（1474.28-1645.21） | 3.10（2.92-3.26） |
|  | Central Latin America | 5104.01（3468.74-7183.12） | 8.02（5.53-11.13） | 78884.74（67579.51-91456.95） | 121.39（104.43-140.28） | 98985.35（93743.16-104614.55） | 157.75（149.44-166.65） | 1536.13（1455.13-1622.43） | 2.50（2.37-2.64） |
|  | Central Sub-Saharan Africa | 2717.10（1968.88-3683.31） | 14.64（10.67-19.76） | 25501.22（22963.32-28050.75） | 133.21（120.29-146.15） | 55182.60（38623.66-76155.73） | 290.47（203.39-401.76） | 858.94（585.87-1203.06） | 4.63（3.16-6.50） |
|  | East Asia | 76992.83(49072.80-114051.66) | 14.31(9.15-21.11) | 853618.95(686587.45-1052347.33) | 155.61(125.32-191.77) | 1708223.78(1442121.52-2066739.55) | 317.04(267.76-383.07) | 26492.31(22044.66-32329.70) | 4.96(4.13-6.05) |
|  | Eastern Europe | 7662.06(4869.17-11243.92) | 8.25(5.20-12.20) | 63054.52(50659.75-77567.18) | 69.51(55.69-85.63) | 132392.10(124205.04-140384.14) | 143.09(134.23-151.78) | 2116.51(1995.11-2225.47) | 2.23(2.13-2.38) |
|  | Eastern Sub-Saharan Africa | 12379.51(8977.11-16609.83) | 19.93(14.51-26.61) | 97197.99(84648.34-111120.35) | 152.62(133.42-174.22) | 291482.52(231799.72-354681.99) | 453.58(361.94-550.96) | 4594.24(3615.71-5634.24) | 7.34(5.79-8.99) |
|  | High-income Asia Pacific | 6976.79(4654.28-9940.31) | 10.29(6.87-14.66) | 97835.41(84548.03-112139.99) | 144.80(125.14-165.96) | 103114.61(88277.02-120264.77) | 151.73(129.85-177.02) | 1513.30(1288.36-1791.73) | 2.22(1.89-2.63) |
|  | High-income North America | 4729.48(2651.72-7619.96) | 4.00(2.21-6.51) | 101237.65(81541.47-124595.25) | 86.65(69.58-106.82) | 74567.07(69002.25-80978.12) | 62.07(57.32-67.55) | 1049.06(1007.02-1091.44) | 0.86(0.83-0.90) |
|  | North Africa and Middle East | 15252.67(11062.67-20486.47) | 12.33(9.02-16.4) | 178789.18(161019.60-197230.58) | 142.19(128.44-156.52) | 461752.77(393040.13-532159.74) | 368.71(313.95-424.48) | 7167.87(6051.13-8303.93) | 5.84(4.94-6.76) |
|  | Oceania | 327.82(239.11-440.55) | 13.69(10.08-18.22) | 3103.75(2760.64-3448.88) | 126.13(112.64-139.67) | 12671.22(8851.23-18045.26) | 531.39(372.52-753.82) | 205.82(140.87-296.30) | 8.78(6.03-12.58) |
|  | South Asia | 40473.45(25953.91-59362.14) | 10.11(6.51-14.72) | 375144.92(300534.21-462846.88) | 92.11(73.98-113.52) | 874356.30(673554.64-1074266.72) | 218.66(168.69-268.04) | 13913.31(10520.46-17265.97) | 3.53(2.67-4.37) |
|  | Southeast Asia | 32943.21(22867.44-45577.69) | 18.25(12.72-25.1) | 297277.24(253828.24-347429.96) | 160.25(137.10-187.16) | 940966.12(834068.46-1062065.63) | 521.00(463.16-586.45) | 15081.62(13317.23-17104.55) | 8.48(7.51-9.59) |
|  | Southern Latin America | 2623.88(1898.73-3518.38) | 14.02(10.18-18.74) | 31867.38(28773.20-34943.31) | 169.92(153.54-186.22) | 45361.31(40889.41-50394.87) | 243.80(219.88-270.75) | 700.16(627.28-780.84) | 3.78(3.39-4.22) |
|  | Southern Sub-Saharan Africa | 2891.30(1917.82-4166.23) | 15.12(10.07-21.61) | 21831.10(17495.31-26996.64) | 110.94(89.21-137.02) | 76732.40(67191.67-87205.05) | 402.12(352.42-456.55) | 1261.34(1099.47-1437.02) | 6.72(5.86-7.65) |
|  | Tropical Latin America | 8808.30(5581.21-13062.43) | 14.82(9.40-21.88) | 68429.55(53999.82-85195.7) | 111.94(88.52-139.20) | 198084.85(187934.28-209208.23) | 334.80(317.73-353.58) | 3327.19(3151.8-3510.04) | 5.68(5.38-5.99) |
|  | Western Europe | 7329.91(4754.89-10845.53) | 5.01(3.23-7.44) | 110402.75(93619.48-128555.68) | 75.44(63.91-87.90) | 133005.21(125216.10-141576.84) | 90.64(85.34-96.48) | 2015.05(1903.30-2128.93) | 1.37(1.29-1.45) |
|  | Western Sub-Saharan Africa | 8856.24(6149.02-12252.51) | 13.85(9.69-18.98) | 118871.55(104542.21-134142.24) | 176.74(155.8-199.06) | 205478.12(165564.58-247877.25) | 317.53(256.98-382.75) | 3185.27(2525.16-3898.16) | 5.04(4.02-6.17) |

Abbreviations: ASR, Age-Standardized Rate; AAPC, Average Annual Percentage Change; DALY, Disability-Adjusted Life Year; CI, Confidence Interval; UI, Uncertainty Interval.

*Numbers in parentheses represent 95% uncertainty intervals (UI)

**Table S2. Country-Specific Burden of ICH Among AYAs in 2021.**

|  | **Incidence** | | | **Prevalence** | | | **DALYs** | | | **Deathes** | | |
| --- | --- | --- | --- | --- | --- | --- | --- | --- | --- | --- | --- | --- |
| Characteristics | Num_2021 (95% UI) | ASR_2021 (95% UI) | AAPCs 1990-2021(95% CI) | Num_2021 (95% UI) | ASR_2021 (95% UI) | AAPCs 1990-2021(95% CI) | Num_2021 (95% UI) | ASR_2021 (95% UI) | AAPCs 1990-2021(95% CI) | Num_2021 (95% UI) | ASR_2021 (95% UI) | AAPCs 1990-2021(95% CI) |
| Afghanistan | 1528.98(1169.36-2001.60) | 15.11(11.68-19.58) | -2.02(-2.11--1.92) | 13693.12(12484.13-14975.48) | 128.25(117.80-139.48) | -1.50(-1.55--1.45) | 50825.73(31989.26-75520.09) | 477.59(301.66-714.48) | -1.35(-1.61--1.08) | 797.31(493.01-1201.51) | 7.76(4.81-11.76) | -1.36(-1.66--1.06) |
| Albania | 70.69(50.91-94.57) | 7.37(5.29-9.89) | -1.22(-1.32--1.13) | 1095.70(1000.4-1196.34) | 114.02(104.06-124.54) | -0.99(-1.02--0.95) | 1148.46(822.72-1553.33) | 119.42(85.45-161.70) | -2.56(-3.87--1.24) | 16.56(11.19-23.32) | 1.72(1.16-2.43) | -2.73(-4.02--1.43) |
| Algeria | 1225.91(871.68-1706.20) | 6.87(4.83-9.64) | -2.46(-2.59--2.33) | 16874.86(15247.83-18611.34) | 95.08(85.75-105.05) | -1.54(-1.59--1.50) | 24747.43(16479.30-36388.26) | 137.17(91.34-201.28) | -2.66(-2.99--2.32) | 383.47(242.94-584.22) | 2.10(1.33-3.19) | -2.80(-3.06--2.55) |
| American Samoa | 2.65(1.96-3.48) | 15.82(11.79-20.72) | -0.68(-0.75--0.61) | 25.55(23.35-27.80) | 456.68(317.83-627.88) | -0.46(-0.48--0.44) | 75.20(52.37-103.38) | 152.80(139.84-166.06) | -0.46(-0.82--0.10) | 1.20(0.81-1.68) | 7.34(4.95-10.25) | -0.45(-0.80--0.10) |
| Andorra | 0.67(0.34-1.15) | 2.48(1.25-4.36) | -0.66(-0.77--0.55) | 14.15(11.92-16.66) | 52.27(43.82-61.75) | -0.11(-0.17--0.06) | 5.45(3.75-7.43) | 19.48(13.51-26.46) | -2.11(-2.51--1.70) | 0.06(0.04-0.10) | 0.21(0.13-0.33) | -2.87(-3.39--2.35) |
| Angola | 1056.93(755.02-1449.45) | 9.55(6.87-13.03) | -1.68(-1.77--1.60) | 11251.24(10093.8-12439.97) | 99.95(90.06-110.08) | -1.15(-1.19--1.12) | 22617.72(14688.91-32694.78) | 204.61(133.54-295.71) | -1.52(-2.17--0.86) | 352.87(222.51-520.10) | 3.26(2.07-4.81) | -1.54(-2.22--0.85) |
| Antigua and Barbuda | 1.78(1.17-2.63) | 5.05(3.28-7.48) | -1.65(-1.71--1.59) | 31.36(27.99-34.96) | 89.37(79.67-99.74) | -0.58(-0.61--0.55) | 20.55(17.07-24.59) | 58.01(48.22-69.37) | -3.95(-5.61--2.25) | 0.30(0.25-0.37) | 0.85(0.70-1.03) | -4.33(-6.15--2.48) |
| Argentina | 1240.44(837.64-1748.02) | 7.00(4.71-9.90) | -2.76(-2.85--2.66) | 19988.07(18175.33-21883.86) | 112.52(102.25-123.28) | -1.63(-1.71--1.56) | 15873.66(13628.58-18422.86) | 88.96(76.36-103.26) | -3.86(-4.82--2.88) | 223.59(190.58-260.74) | 1.25(1.06-1.46) | -4.24(-5.37--3.09) |
| Armenia | 52.76(31.38-83.02) | 4.52(2.62-7.29) | -1.21(-1.36--1.07) | 711.62(614.62-809.26) | 60.56(52.02-69.23) | -0.45(-0.49--0.42) | 421.25(349.95-506.51) | 34.39(28.43-41.42) | -3.73(-4.94--2.50) | 5.13(4.31-5.98) | 0.40(0.34-0.47) | -4.54(-5.86--3.21) |
| Australia | 190.69(100.48-320.86) | 2.11(1.09-3.60) | -1.73(-1.84--1.63) | 4025.34(3340.42-4795.48) | 44.51(36.82-53.15) | -0.77(-0.82--0.72) | 1751.95(1446.49-2086.81) | 18.67(15.38-22.27) | -2.23(-2.94--1.50) | 20.66(16.91-25.16) | 0.21(0.17-0.26) | -2.76(-3.43--2.08) |
| Austria | 87.31(44.56-149.44) | 2.98(1.50-5.16) | -1.72(-1.89--1.54) | 1857.41(1603.62-2131.60) | 62.94(54.20-72.32) | -0.67(-0.69--0.65) | 631.13(528.31-750.11) | 20.93(17.49-24.90) | -3.89(-4.42--3.35) | 6.72(5.59-7.98) | 0.22(0.18-0.26) | -4.91(-5.70--4.13) |
| Azerbaijan | 327.66(233.02-455.18) | 7.14(5.00-10.04) | -2.03(-2.27--1.79) | 3540.59(3138.49-3960.21) | 77.55(68.38-87.13) | -1.13(-1.16--1.10) | 4663.51(3073.24-6482.66) | 101.17(66.68-140.48) | -3.04(-3.95--2.12) | 69.87(43.47-100.92) | 1.49(0.93-2.15) | -3.33(-4.21--2.45) |
| Bahamas | 11.56(8.69-15.28) | 7.43(5.58-9.82) | -1.17(-1.23--1.12) | 150.76(135.82-167.88) | 97.21(87.56-108.27) | -0.49(-0.53--0.46) | 202.63(153.69-258.11) | 130.03(98.64-165.63) | -2.06(-2.87--1.25) | 3.34(2.50-4.32) | 2.14(1.60-2.77) | -2.11(-2.96--1.25) |
| Bahrain | 41.72(26.59-62.19) | 5.80(3.64-8.74) | -1.68(-1.97--1.38) | 835.47(758.94-918.23) | 113.6(103.02-125.01) | -1.14(-1.16--1.12) | 902.72(663.23-1211.01) | 122.06(89.79-163.34) | -1.76(-2.48--1.03) | 13.24(9.25-18.33) | 1.76(1.23-2.43) | -1.74(-2.40--1.09) |
| Bangladesh | 7359.69(5277.70-10050.48) | 10.91(7.83-14.89) | -1.31(-1.34--1.28) | 67582.24(60757.89-74587.88) | 99.8(89.81-110.07) | -0.62(-0.63--0.61) | 209770.34(126809.97-306507.08) | 310.42(187.99-453.25) | -1.55(-1.96--1.13) | 3420.04(2010.61-5053.12) | 5.08(2.99-7.50) | -1.59(-2.01--1.17) |
| Barbados | 5.04(3.35-7.33) | 4.92(3.24-7.20) | -1.41(-1.53--1.29) | 90.81(80.96-101.02) | 90.02(80.18-100.22) | -0.45(-0.49--0.42) | 72.88(56.68-95.15) | 69.94(54.41-91.26) | -2.31(-3.21--1.39) | 1.14(0.86-1.52) | 1.08(0.81-1.44) | -2.51(-3.70--1.30) |
| Belarus | 245.30(177.22-334.02) | 6.87(4.80-9.63) | -1.14(-1.33--0.95) | 2185.54(1950.20-2438.30) | 65.64(58.16-73.72) | -0.41(-0.43--0.39) | 4123.42(3275.59-5123.83) | 108.08(85.83-134.20) | -1.47(-2.38--0.56) | 68.01(53.06-85.69) | 1.73(1.35-2.19) | -1.50(-2.47--0.53) |
| Belgium | 82.97(43.22-140.98) | 2.30(1.18-3.96) | -2.7(-2.79--2.60) | 1973.19(1632.30-2335.01) | 54.38(44.86-64.47) | -0.79(-0.81--0.77) | 853.75(721.18-999.64) | 22.96(19.39-26.90) | -5.26(-5.90--4.60) | 10.34(8.81-12.04) | 0.27(0.23-0.32) | -6.10(-7.17--5.01) |
| Belize | 10.48(7.37-14.56) | 5.79(4.11-7.98) | -0.58(-0.65--0.51) | 150.42(133.07-169.34) | 82.00(72.68-92.17) | -0.48(-0.52--0.44) | 184.99(152.00-220.14) | 104.12(85.51-123.94) | -1.72(-2.79--0.63) | 2.95(2.40-3.55) | 1.68(1.37-2.02) | -1.68(-2.83--0.50) |
| Benin | 495.13(345.59-688.37) | 10.57(7.47-14.58) | -0.81(-0.88--0.74) | 7240.93(6684.41-7842.38) | 147.27(136.26-159.15) | -0.61(-0.63--0.60) | 9602.02(6404-13415.49) | 203.80(136.59-284.18) | -0.83(-0.99--0.67) | 145.02(92.95-208.02) | 3.16(2.04-4.52) | -0.87(-1.04--0.70) |
| Bermuda | 0.75(0.41-1.24) | 4.06(2.18-6.82) | -1.14(-1.22--1.06) | 15.93(14.25-17.69) | 86.67(77.30-96.37) | -0.49(-0.54--0.44) | 4.8(3.86-6.02) | 24.34(19.58-30.56) | -3.45(-3.77--3.14) | 0.06(0.05-0.08) | 0.29(0.22-0.39) | -4.16(-4.59--3.73) |
| Bhutan | 16.64(10.27-25.16) | 4.77(2.93-7.23) | -1.69(-1.73--1.65) | 215.23(186.16-246.07) | 61.47(53.13-70.32) | -0.80(-0.83--0.77) | 301.18(168.27-476.22) | 86.07(48.12-136.03) | -2.08(-2.28--1.88) | 4.65(2.42-7.69) | 1.33(0.69-2.19) | -2.21(-2.42--2.01) |
| Bolivia (Plurinational State of) | 252.12(169.62-359.70) | 5.18(3.48-7.37) | -2.91(-2.97--2.85) | 3807.92(3332.70-4320.58) | 78.06(68.36-88.54) | -1.66(-1.71--1.60) | 6925.98(4144.85-10472.75) | 142.44(85.33-215.13) | -3.53(-3.68--3.39) | 108.76(62.95-166.74) | 2.25(1.30-3.44) | -3.60(-3.73--3.46) |
| Bosnia and Herzegovina | 38.07(23.25-59.01) | 3.59(2.15-5.65) | -1.08(-1.29--0.87) | 707.32(620.68-796.28) | 67.44(59.05-76.06) | -0.61(-0.62--0.59) | 535.31(374.20-714.02) | 49.26(34.67-65.62) | -2.72(-3.62--1.81) | 7.04(4.42-10.09) | 0.63(0.40-0.90) | -3.23(-4.35--2.09) |
| Botswana | 100.96(73.46-135.97) | 9.17(6.66-12.36) | -1.14(-1.20--1.07) | 975.57(876.06-1074.34) | 89.39(80.15-98.58) | -0.53(-0.57--0.49) | 1469.54(763.61-2293.94) | 134.05(69.93-209.25) | -2.85(-3.80--1.88) | 23.00(10.98-37.04) | 2.08(1.00-3.36) | -2.95(-3.98--1.91) |
| Brazil | 4427.96(2833.96-6453.45) | 4.95(3.15-7.25) | -3.55(-3.62--3.48) | 56711.86(46711.96-68178.87) | 64.12(52.71-77.19) | -1.82(-1.86--1.77) | 96765.96(90879.58-102663.43) | 107.33(100.76-113.89) | -3.59(-4.00--3.18) | 1591.51(1496.68-1689.24) | 1.75(1.64-1.86) | -3.72(-4.14--3.30) |
| Brunei Darussalam | 12.39(8.09-18.47) | 5.75(3.69-8.67) | -2.94(-3.01--2.86) | 203.41(182.71-225.61) | 95.53(85.59-106.16) | -1.70(-1.75--1.66) | 255.21(187.42-334.12) | 113.53(83.30-148.79) | -2.58(-3.12--2.04) | 3.99(2.80-5.42) | 1.75(1.23-2.38) | -2.57(-3.19--1.94) |
| Bulgaria | 173.01(134.48-223.01) | 7.90(5.99-10.39) | -2.28(-2.34--2.22) | 1936.89(1727.30-2167.66) | 93.05(82.48-104.79) | -1.49(-1.52--1.46) | 3969.89(3261.53-4707.34) | 182.56(149.99-216.58) | -2.33(-2.91--1.74) | 63.56(52.00-76.62) | 2.85(2.33-3.43) | -2.39(-3.01--1.76) |
| Burkina Faso | 740.63(523.08-1026.64) | 9.43(6.76-12.93) | -0.52(-0.57--0.46) | 10429.30(9566.14-11334.01) | 127.20(116.89-137.96) | -0.47(-0.49--0.45) | 15313.72(9972.58-21951.22) | 194.85(127.24-278.24) | -0.65(-0.92--0.38) | 235.62(146.41-343.84) | 3.06(1.91-4.45) | -0.70(-0.98--0.41) |
| Burundi | 623.48(469.39-820.72) | 12.70(9.59-16.69) | -2.66(-2.75--2.58) | 5385.88(4859.41-5936.05) | 108.88(98.52-119.64) | -1.83(-1.91--1.76) | 15887.34(10311.71-22720) | 318.45(207.50-454.50) | -2.70(-2.86--2.54) | 252.15(159.63-364.98) | 5.13(3.26-7.41) | -2.71(-2.87--2.54) |
| Cabo Verde | 24.09(16.75-33.30) | 9.45(6.54-13.10) | -2.03(-2.12--1.95) | 412.75(378.88-447.75) | 162.24(148.85-176.09) | -1.07(-1.10--1.04) | 385.38(254.51-542.36) | 151.27(100.06-212.60) | -3.11(-3.59--2.63) | 5.65(3.46-8.34) | 2.21(1.35-3.25) | -3.37(-3.93--2.81) |
| Cambodia | 921.85(697.96-1210.41) | 12.71(9.61-16.69) | -0.59(-0.70--0.48) | 8212.52(7433.26-9021.10) | 112.94(102.22-124.06) | -0.34(-0.38--0.29) | 18978.64(12900.90-28369.58) | 261.60(177.87-390.69) | -1.97(-2.10--1.85) | 301.95(198.35-462.80) | 4.16(2.74-6.38) | -2.08(-2.21--1.96) |
| Cameroon | 1621.46(1203.65-2164.11) | 13.66(10.20-18.12) | -0.46(-0.73--0.20) | 20428.85(18927.23-22076.34) | 166.84(154.82-179.98) | -0.25(-0.32--0.19) | 37408.63(22895.21-55159.22) | 312.81(191.95-460.95) | -0.67(-0.83--0.51) | 582.23(340.10-881.83) | 4.96(2.91-7.50) | -0.70(-0.87--0.53) |
| Canada | 374.36(211.51-593.50) | 3.01(1.68-4.84) | -0.74(-0.80--0.67) | 8114.51(7015.79-9259.94) | 65.73(56.69-75.22) | -0.56(-0.59--0.53) | 3724.24(3094.93-4433.49) | 29.23(24.28-34.82) | -0.98(-1.52--0.45) | 44.82(37.09-53.73) | 0.34(0.28-0.41) | -1.04(-1.77--0.31) |
| Central African Republic | 276.97(212.96-358.92) | 14.02(10.85-18.13) | -0.74(-0.85--0.63) | 2348.17(2145.34-2560.23) | 116.18(106.46-126.26) | -0.60(-0.62--0.59) | 6914.73(3949.81-10908.28) | 346.43(196.70-548.55) | -0.66(-1.05--0.27) | 110.53(60.89-177.34) | 5.66(3.10-9.11) | -0.64(-1.03--0.24) |
| Chad | 774.04(574.19-1026.15) | 14.18(10.64-18.61) | -0.15(-0.27--0.03) | 10064.79(9335.90-10865.01) | 174.70(162.42-188.17) | -0.19(-0.22--0.16) | 17633.43(11652.11-26252.62) | 315.03(208.72-469.64) | -0.11(-0.44-0.22) | 268.51(171.75-409.20) | 4.96(3.18-7.56) | -0.13(-0.48-0.23) |
| Chile | 438.98(267.99-674.15) | 6.10(3.68-9.41) | -1.28(-1.33--1.23) | 8494.20(7637.06-9395.02) | 116.85(104.97-129.38) | -0.60(-0.63--0.56) | 4459.74(3764.35-5227.25) | 60.95(51.45-71.45) | -2.74(-3.37--2.11) | 56.10(47.34-65.89) | 0.76(0.64-0.89) | -3.27(-4.04--2.49) |
| China | 49364.51(33444.57-69293.18) | 9.44(6.33-13.41) | -1.35(-1.42--1.28) | 560073.62(465399.54-672628.39) | 110.26(91.31-132.64) | -1.11(-1.16--1.06) | 1102003.76(914613.09-1305524.73) | 208.12(172.46-247.04) | -1.28(-1.65--0.91) | 17518.35(14226.03-20987.98) | 3.24(2.63-3.89) | -1.28(-1.72--0.84) |
| Colombia | 680.52(399.19-1090.66) | 3.39(1.98-5.43) | -2.57(-2.64--2.50) | 13926.07(12210.04-15763.77) | 68.99(60.49-78.11) | -1.55(-1.58--1.52) | 9248.94(7419.73-11272.55) | 45.90(36.81-55.95) | -4.00(-4.86--3.13) | 136.32(106.41-169.29) | 0.68(0.53-0.84) | -4.21(-5.22--3.19) |
| Comoros | 29.83(20.87-40.98) | 10.07(7.07-13.81) | -2.18(-2.35--2.01) | 299.56(270.28-331.48) | 100.23(90.60-110.74) | -1.50(-1.55--1.45) | 694.97(460.98-1000.00) | 232.84(154.42-334.8) | -2.48(-4.77--0.13) | 10.98(7.10-15.98) | 3.71(2.40-5.40) | -2.52(-4.87--0.12) |
| Congo | 199.75(144.09-272.11) | 9.37(6.79-12.72) | -2.41(-2.47--2.35) | 2136.16(1925.82-2349.74) | 99.34(89.69-109.10) | -1.45(-1.49--1.41) | 5064.74(3139.78-7907.24) | 237.41(146.64-372.13) | -1.54(-2.04--1.03) | 81.31(48.33-130.64) | 3.84(2.27-6.19) | -1.55(-2.08--1.03) |
| Cook Islands | 0.75(0.55-1.03) | 13.15(9.65-17.92) | -1.01(-1.08--0.93) | 8.17(7.48-8.94) | 142.19(130.20-155.38) | -0.54(-0.56--0.51) | 14.40(9.59-20.91) | 251.79(167.57-365.94) | -1.86(-2.01--1.71) | 0.22(0.14-0.33) | 3.85(2.42-5.81) | -1.98(-2.14--1.81) |
| Costa Rica | 79.81(47.51-126.50) | 4.12(2.43-6.57) | -1.26(-1.31--1.20) | 1748.36(1571.25-1934.36) | 90.09(80.89-99.75) | -0.70(-0.74--0.66) | 755.10(624.11-905.90) | 38.01(31.44-45.58) | -2.18(-2.76--1.60) | 10.52(8.49-12.77) | 0.52(0.42-0.64) | -2.44(-3.14--1.73) |
| Coted'Ivoire | 1320.13(969.56-1756.10) | 12.31(9.09-16.31) | -1.02(-1.17--0.86) | 19330.61(17923.35-20792.48) | 177.33(164.55-190.61) | -0.62(-0.64--0.61) | 31225.39(19946.27-45560.88) | 290.85(185.98-424.33) | -0.75(-1.06--0.44) | 482.64(291.34-720.95) | 4.55(2.75-6.79) | -0.75(-1.08--0.42) |
| Croatia | 38.82(22.69-61.54) | 2.96(1.68-4.79) | -2.93(-2.97--2.89) | 827.56(712.83-954.92) | 63.26(54.3-73.19) | -1.44(-1.52--1.36) | 411.90(331.93-508.07) | 30.01(24.16-37.04) | -4.87(-5.54--4.18) | 4.59(3.67-5.71) | 0.32(0.26-0.40) | -6.04(-7.48--4.59) |
| Cuba | 169.76(109.21-253.93) | 4.53(2.87-6.86) | -2.05(-2.16--1.93) | 2993.84(2660.84-3360.76) | 81.06(71.92-91.11) | -0.85(-0.88--0.82) | 2577.86(2139.26-3095.78) | 67.8(56.25-81.39) | -2.54(-4.00--1.07) | 40.58(33.26-49.24) | 1.06(0.87-1.28) | -2.66(-4.25--1.04) |
| Cyprus | 16.43(9.03-26.99) | 3.04(1.61-5.12) | -1.29(-1.33--1.25) | 351.84(304.53-403.51) | 64.39(55.33-74.10) | -0.92(-0.95--0.89) | 134.18(97.46-176.84) | 23.34(16.86-31.02) | -3.07(-3.94--2.19) | 1.55(1.01-2.21) | 0.26(0.16-0.37) | -3.84(-4.95--2.71) |
| Czechia | 110.13(60.29-181.7) | 3.53(1.89-5.93) | -1.90(-2.11--1.69) | 2073.15(1839.49-2324.86) | 66.10(58.42-74.29) | -1.05(-1.08--1.02) | 939.89(738.58-1179.26) | 28.36(22.31-35.54) | -3.81(-4.40--3.21) | 9.88(7.81-12.45) | 0.28(0.22-0.35) | -5.11(-6.11--4.09) |
| Democratic People's Republic of Korea | 1747.82(1328.31-2267.36) | 16.52(12.51-21.47) | -0.42(-0.43--0.40) | 15832.19(14597.16-17160.85) | 150.91(138.93-163.8) | -0.70(-0.73--0.68) | 50552.01(32408.71-79837.80) | 475.14(305.07-748.75) | 0.33(0.25-0.41) | 827.16(514.28-1336.34) | 7.72(4.8-12.44) | 0.44(0.35-0.53) |
| Democratic Republic of the Congo | 3356.56(2471.44-4503.09) | 10.29(7.62-13.70) | -0.93(-0.99--0.86) | 31570.77(28318.44-35179.45) | 94.56(85.22-104.87) | -1.00(-1.02--0.97) | 62306.02(37772.84-96883.10) | 189.16(114.91-293.95) | -1.07(-1.20--0.93) | 972.50(568.76-1547.81) | 3.02(1.77-4.80) | -1.04(-1.18--0.90) |
| Denmark | 47.75(23.82-84.33) | 2.59(1.28-4.59) | -1.16(-1.43--0.89) | 1078.27(915.22-1254.06) | 58.29(49.44-67.82) | -0.59(-0.64--0.53) | 395.20(328.49-473.63) | 21.31(17.70-25.54) | -3.84(-5.10--2.57) | 4.37(3.57-5.31) | 0.24(0.19-0.29) | -4.69(-6.36--2.99) |
| Djibouti | 53.03(37.50-73.46) | 9.75(6.89-13.52) | -1.68(-1.76--1.61) | 563.94(507.80-620.67) | 103.73(93.38-114.18) | -1.07(-1.10--1.04) | 1114.49(666.03-1769.69) | 204.43(122.17-324.57) | -1.51(-1.91--1.12) | 17.77(10.17-28.82) | 3.26(1.86-5.28) | -1.54(-1.94--1.13) |
| Dominica | 1.25(0.83-1.83) | 4.80(3.18-7.05) | -1.55(-1.61--1.48) | 20.17(17.88-22.67) | 77.74(68.91-87.40) | -0.71(-0.74--0.68) | 24.74(17.37-34.25) | 95.03(66.74-131.59) | -0.78(-1.12--0.43) | 0.40(0.27-0.56) | 1.52(1.04-2.15) | -0.75(-1.12--0.37) |
| Dominican Republic | 417.77(311.48-551.92) | 9.29(6.92-12.26) | -0.40(-0.42--0.37) | 4294.83(3880.32-4768.27) | 94.84(85.72-105.26) | -0.47(-0.52--0.43) | 9229.67(6392.31-13276.03) | 204.15(141.48-293.57) | -1.10(-2.70-0.53) | 150.33(102.70-218.54) | 3.33(2.28-4.84) | -1.14(-2.78-0.52) |
| Ecuador | 412.5(287.29-576.43) | 5.69(3.97-7.94) | -2.26(-2.34--2.18) | 5917.91(5270.34-6634.23) | 81.57(72.68-91.42) | -1.06(-1.10--1.02) | 6336.14(4913.61-7996.60) | 87.67(67.96-110.7) | -4.06(-5.02--3.10) | 97.53(73.77-125.29) | 1.35(1.02-1.74) | -4.22(-5.22--3.22) |
| Egypt | 2401.13(1649.16-3380.12) | 5.74(3.95-8.06) | -1.66(-1.75--1.56) | 34469.57(30404.41-38706.72) | 82.40(72.72-92.48) | -0.85(-0.88--0.82) | 76509.89(49692.88-108791.44) | 182.56(118.6-259.61) | -3.01(-3.63--2.38) | 1182.31(748.21-1713.41) | 2.83(1.79-4.10) | -3.12(-3.79--2.45) |
| El Salvador | 111.52(73.97-162.58) | 4.42(2.95-6.41) | -3.33(-3.48--3.18) | 1949.12(1714.34-2194.11) | 76.71(67.58-86.27) | -2.21(-2.31--2.10) | 2388.21(1630.35-3339.37) | 96.10(65.55-134.4) | -3.09(-4.82--1.33) | 37.22(24.55-53.25) | 1.52(1.00-2.17) | -3.40(-5.05--1.73) |
| Equatorial Guinea | 40.98(27.38-58.95) | 6.28(4.22-9.01) | -3.18(-3.29--3.08) | 592.11(529.08-656.40) | 90.08(80.70-99.59) | -1.45(-1.47--1.42) | 940.62(507.73-1573.29) | 148.00(79.76-247.53) | -3.03(-3.64--2.41) | 14.37(7.03-24.65) | 2.32(1.13-3.97) | -3.13(-3.79--2.46) |
| Eritrea | 390.27(290.67-518.47) | 14.96(11.17-19.84) | -1.43(-1.52--1.33) | 3068.35(2791.29-3357.20) | 116.02(105.83-126.64) | -1.10(-1.12--1.07) | 9881.58(5933.40-15401.80) | 374.96(224.59-586.10) | -1.84(-2.19--1.50) | 159.45(93.61-252.67) | 6.13(3.59-9.74) | -1.86(-2.20--1.51) |
| Estonia | 14.79(8.06-24.65) | 3.44(1.83-5.86) | -2.69(-3.02--2.37) | 190.81(164.29-219.35) | 44.42(38.07-51.27) | -1.05(-1.07--1.02) | 125.41(101.31-155.55) | 27.06(21.77-33.59) | -4.00(-5.08--2.91) | 1.62(1.28-2.05) | 0.33(0.26-0.42) | -4.68(-6.09--3.25) |
| Eswatini | 45.88(34.37-60.61) | 9.53(7.15-12.57) | 0.20(0.09-0.32) | 408.28(361.25-458.40) | 83.4(74.02-93.41) | 0.07(-0.03-0.17) | 1394.07(777.90-2207.09) | 286.69(159.84-454.49) | 0.48(0.22-0.74) | 22.5(12.12-36.20) | 4.68(2.52-7.54) | 0.52(0.24-0.79) |
| Ethiopia | 3141.8(2066.93-4524.78) | 7.54(4.99-10.78) | -3.70(-3.86--3.55) | 35149.73(28781.25-42418.77) | 82.91(68.05-99.91) | -2.26(-2.31--2.21) | 75758.58(54798.16-97852.45) | 179.10(129.84-230.50) | -3.15(-3.35--2.95) | 1174.14(831.92-1534.41) | 2.84(2.02-3.70) | -3.20(-3.40--2.99) |
| Fiji | 48.25(35.91-63.54) | 13.45(10.01-17.71) | -1.26(-1.30--1.22) | 518.62(475.03-563.70) | 144.60(132.42-157.21) | -0.82(-0.84--0.79) | 1540.25(1090.29-2077.52) | 429.31(303.94-578.90) | -1.11(-1.35--0.86) | 24.35(16.83-33.41) | 6.77(4.68-9.29) | -1.18(-1.49--0.87) |
| Finland | 62.58(37.40-96.12) | 3.59(2.11-5.58) | -0.68(-0.82--0.54) | 1162.96(1013.38-1333.40) | 66.77(57.97-76.72) | -0.26(-0.30--0.23) | 461.88(385.19-547.50) | 26.00(21.68-30.84) | -3.87(-5.21--2.52) | 5.39(4.41-6.49) | 0.30(0.24-0.36) | -4.97(-6.76--3.14) |
| France | 478.83(259.4-782.42) | 2.37(1.27-3.89) | -1.83(-1.95--1.71) | 10236.36(8554.44-12075.27) | 50.52(42.15-59.65) | -0.55(-0.60--0.50) | 4513.53(3751.79-5329.57) | 21.90(18.19-25.88) | -3.90(-4.73--3.07) | 54.76(44.60-66.54) | 0.26(0.21-0.32) | -4.66(-5.77--3.54) |
| Gabon | 58.96(41.68-81.58) | 8.28(5.88-11.45) | -1.93(-1.97--1.88) | 722.68(652.59-794.17) | 100.73(91.17-110.46) | -1.17(-1.22--1.13) | 1059.27(620.99-1651.70) | 151.34(88.35-236.42) | -1.82(-2.11--1.52) | 16.36(8.96-26.36) | 2.37(1.30-3.83) | -1.86(-2.17--1.55) |
| Gambia | 106.61(75.97-146.36) | 11.77(8.47-16.05) | -0.55(-0.72--0.38) | 1382.48(1267.40-1505.96) | 147.60(135.63-160.40) | -0.78(-0.80--0.75) | 3027.45(1967.87-4487.43) | 327.80(214.22-484.69) | -0.42(-1.36-0.52) | 47.06(29.95-70.80) | 5.21(3.34-7.82) | -0.41(-1.40-0.58) |
| Georgia | 145.99(110.46-190.54) | 11.56(8.61-15.29) | -2.72(-3.00--2.44) | 1426.26(1296.41-1555.96) | 115.38(104.50-126.24) | -2.02(-2.09--1.96) | 1661.92(1333.95-2012.64) | 128.87(103.40-156.15) | -3.84(-6.28--1.33) | 25.06(19.80-31) | 1.90(1.50-2.36) | -3.92(-6.55--1.21) |
| Germany | 706.82(370.38-1204.92) | 2.67(1.37-4.62) | -2.01(-2.14--1.87) | 14938.80(12729.67-17286.19) | 56.06(47.59-65.01) | -0.96(-1.01--0.91) | 6714.65(5650.52-7882.14) | 24.43(20.55-28.70) | -4.31(-5.20--3.41) | 82.67(69.11-98.13) | 0.29(0.24-0.35) | -5.03(-6.11--3.92) |
| Ghana | 2215.49(1644.38-2933.79) | 16.32(12.16-21.55) | -1.12(-1.19--1.05) | 27162.08(25046.22-29353.88) | 196.39(181.25-212.10) | -0.68(-0.71--0.65) | 49879.30(34161.51-69154.54) | 367.94(252.45-509.19) | -1.71(-1.88--1.54) | 786.93(525.01-1111.61) | 5.87(3.93-8.27) | -1.76(-1.93--1.58) |
| Greece | 134.60(87.59-196.87) | 4.52(2.88-6.72) | -2.28(-2.40--2.17) | 2231.63(1959.14-2530.32) | 75.32(65.82-85.68) | -1.13(-1.16--1.09) | 1879.79(1603.93-2164.72) | 62.25(53.06-71.72) | -3.40(-4.11--2.68) | 27.46(23.14-32.07) | 0.89(0.75-1.04) | -3.64(-4.43--2.85) |
| Greenland | 1.01(0.68-1.45) | 4.71(3.14-6.83) | -2.51(-2.62--2.39) | 17.16(14.95-19.61) | 81.38(70.68-93.17) | -1.46(-1.54--1.37) | 18.66(12.30-25.64) | 85.76(56.63-117.68) | -3.37(-3.53--3.21) | 0.29(0.18-0.41) | 1.30(0.80-1.85) | -3.53(-3.71--3.35) |
| Grenada | 2.34(1.67-3.24) | 6.03(4.33-8.28) | -2.39(-2.47--2.30) | 35.31(31.35-39.72) | 88.74(78.90-99.71) | -0.85(-0.87--0.82) | 41.20(32.46-50.72) | 107.89(84.98-132.81) | -4.01(-4.63--3.39) | 0.67(0.52-0.83) | 1.77(1.38-2.20) | -4.07(-4.72--3.43) |
| Guam | 6.88(5.14-9.06) | 12.56(9.4-16.51) | 0.33(0.26-0.41) | 72.82(66.76-79.55) | 132.37(121.41-144.55) | 0.02(0.02-0.03) | 138.46(105.76-180.23) | 252.91(193.48-328.62) | 0.38(-0.82-1.60) | 2.14(1.60-2.84) | 3.92(2.94-5.20) | 0.72(-0.59-2.05) |
| Guatemala | 559.4(431.89-721.15) | 8.71(6.80-11.12) | -2.31(-2.63--1.98) | 6605.99(5946.52-7290.46) | 102.09(92.18-112.37) | -1.57(-1.63--1.51) | 9930.41(8086.62-12053.82) | 156.46(127.21-190.10) | -2.17(-3.36--0.97) | 156.73(125.95-192.25) | 2.51(2.01-3.08) | -2.17(-3.42--0.91) |
| Guinea | 718.99(535.79-947.19) | 15.28(11.45-20.02) | -0.26(-0.36--0.16) | 8834.75(8205.83-9508.09) | 181.73(169.04-195.28) | -0.32(-0.33--0.32) | 16197.93(10431.41-23497.41) | 338.83(218.21-489.60) | -0.32(-0.58--0.07) | 249.57(155.25-368.49) | 5.33(3.32-7.83) | -0.35(-0.61--0.08) |
| Guinea-Bissau | 134.90(103.45-174.72) | 17.47(13.49-22.49) | -1.08(-1.16--1.01) | 1410.78(1308.09-1519.26) | 176.76(164.20-190.11) | -1.05(-1.10--1.01) | 4209.63(2822.87-6105.28) | 536.26(359.14-780.90) | -1.19(-1.32--1.05) | 67.31(44.28-99.01) | 8.72(5.74-12.87) | -1.18(-1.30--1.07) |
| Guyana | 32.35(25.43-40.81) | 11.04(8.72-13.86) | -1.85(-1.97--1.73) | 331.61(301.36-364.45) | 111.02(101.17-121.77) | -1.36(-1.41--1.31) | 808.00(597.88-1083.49) | 276.93(204.93-370.79) | -1.79(-2.86--0.71) | 13.27(9.72-17.99) | 4.61(3.38-6.23) | -1.78(-2.95--0.61) |
| Haiti | 658.63(506.56-849.60) | 12.25(9.43-15.78) | -1.51(-1.57--1.45) | 6033.71(5480.56-6631.56) | 111.24(101.12-122.18) | -0.94(-0.99--0.90) | 22037.13(13934.20-33418.29) | 407.94(258.18-618.25) | -1.59(-2.01--1.16) | 365.50(228.88-557.33) | 6.79(4.25-10.34) | -1.61(-2.03--1.19) |
| Honduras | 156.65(93.65-244.79) | 3.72(2.26-5.73) | -2.59(-2.67--2.51) | 3246.13(2842.37-3690.94) | 76.16(66.81-86.52) | -1.32(-1.35--1.29) | 5972.01(2961.96-9670.79) | 146.52(72.81-237.09) | -3.35(-3.90--2.79) | 96.21(45.32-158.62) | 2.40(1.14-3.95) | -3.38(-3.95--2.81) |
| Hungary | 110.66(65.81-174.93) | 3.85(2.24-6.19) | -3.44(-3.57--3.32) | 2109.51(1866.13-2353.79) | 73.51(64.89-82.17) | -1.40(-1.44--1.36) | 1418.51(1141.34-1742.25) | 46.86(37.7-57.56) | -4.91(-6.35--3.45) | 18.31(14.61-22.69) | 0.59(0.47-0.72) | -5.67(-7.62--3.69) |
| Iceland | 3.39(1.69-5.96) | 2.77(1.37-4.90) | -1.52(-1.76--1.28) | 70.86(61.02-81.76) | 57.55(49.48-66.46) | -0.68(-0.71--0.65) | 26.57(21.81-32.17) | 21.19(17.39-25.66) | -3.38(-3.82--2.93) | 0.30(0.24-0.36) | 0.23(0.19-0.29) | -4.23(-5.15--3.31) |
| India | 42028.83(27190.11-61474.61) | 7.00(4.53-10.22) | -0.90(-0.99--0.81) | 427987.61(348127.73-518266.15) | 70.97(57.75-85.93) | -0.68(-0.72--0.63) | 684599.65(556843.63-820558.67) | 114.11(92.85-136.76) | -1.58(-2.11--1.04) | 10800.82(8659.26-13142.28) | 1.81(1.45-2.20) | -1.62(-2.21--1.04) |
| Indonesia | 16942.45(11571.27-23535.65) | 14.52(9.90-20.19) | -1.72(-1.76--1.68) | 159247.47(130201.55-192578.33) | 137.02(111.99-165.72) | -1.19(-1.23--1.16) | 508945.94(410426.95-673631.99) | 436.44(351.9-577.49) | -1.33(-1.42--1.24) | 8294.54(6600.67-11126.93) | 7.09(5.64-9.51) | -1.34(-1.44--1.24) |
| Iran (Islamic Republic of) | 1485.29(949.42-2175.02) | 4.11(2.60-6.08) | -1.48(-1.53--1.43) | 26307.17(21947.34-31447.93) | 70.79(58.72-84.81) | -1.05(-1.07--1.02) | 31525.18(28226.84-35142.43) | 86.27(76.97-96.33) | -1.59(-1.69--1.49) | 468.90(416.81-525.87) | 1.25(1.11-1.40) | -1.70(-1.82--1.58) |
| Iraq | 2195.11(1673.70-2843.98) | 12.98(9.92-16.78) | -1.03(-1.07--0.99) | 25112.78(23162.36-27099.72) | 148.89(137.51-160.5) | -0.80(-0.84--0.77) | 42558.79(29458.61-61691.82) | 251.97(174.72-365.74) | -2.39(-2.63--2.16) | 644.54(432.06-968.65) | 3.86(2.59-5.81) | -2.52(-2.78--2.26) |
| Ireland | 44.54(22.48-77.54) | 2.78(1.39-4.89) | -1.50(-1.62--1.38) | 968.07(834.45-1112.45) | 60.08(51.66-69.12) | -0.65(-0.68--0.62) | 254.32(201.65-315.62) | 15.41(12.23-19.11) | -3.88(-4.34--3.42) | 2.23(1.79-2.70) | 0.13(0.10-0.16) | -5.37(-5.80--4.94) |
| Israel | 67.02(32.02-120.77) | 2.01(0.96-3.63) | -1.80(-1.91--1.70) | 1579.63(1286.83-1895.17) | 47.53(38.72-57.02) | -0.61(-0.63--0.59) | 646.45(531.40-771.31) | 19.36(15.91-23.09) | -4.37(-5.31--3.42) | 7.50(6.05-9.06) | 0.22(0.18-0.27) | -5.11(-6.34--3.86) |
| Italy | 447.54(252.29-730.98) | 2.76(1.53-4.57) | -2.28(-2.32--2.23) | 9313.50(7622.06-11257.81) | 56.57(46.13-68.44) | -1.39(-1.42--1.36) | 5674.54(5107.62-6283.66) | 33.69(30.26-37.37) | -3.59(-4.46--2.71) | 76.61(71.01-82.78) | 0.45(0.41-0.48) | -4.03(-5.09--2.96) |
| Jamaica | 74.26(52.93-102.79) | 6.26(4.46-8.68) | -0.64(-0.71--0.56) | 1109.33(991.45-1235.33) | 92.75(82.89-103.27) | -0.60(-0.62--0.57) | 1308.83(963.08-1778.25) | 109.79(80.84-149.08) | -1.28(-2.53--0.03) | 21.12(15.10-29.27) | 1.78(1.27-2.46) | -1.21(-2.59-0.19) |
| Japan | 1580.46(919.6-2529.38) | 4.57(2.61-7.44) | -0.95(-1.13--0.76) | 30141.20(24942.53-36019.69) | 88.59(73.04-106.09) | -0.36(-0.44--0.29) | 18778.79(16969.92-20885.07) | 52.41(47.11-58.56) | -1.44(-1.80--1.07) | 246.63(235.66-258.64) | 0.67(0.64-0.70) | -1.80(-2.66--0.94) |
| Jordan | 235.97(140.32-375.42) | 4.45(2.67-7.03) | -1.90(-2.07--1.73) | 4580.94(4078.84-5102.15) | 87.00(77.55-96.84) | -0.99(-1.02--0.96) | 5195.19(3835.23-6846.21) | 98.74(72.75-130.29) | -3.47(-4.02--2.92) | 74.57(52.89-100.70) | 1.43(1.01-1.93) | -3.70(-4.28--3.12) |
| Kazakhstan | 692.54(520.63-911.22) | 9.17(6.8-12.22) | -1.33(-1.78--0.87) | 6721.45(6022.30-7414.45) | 90.94(81.21-100.58) | -1.29(-1.36--1.21) | 9507.04(7140.65-11795.77) | 123.95(93.20-153.71) | -2.00(-2.62--1.37) | 147.77(106.30-187.31) | 1.90(1.37-2.41) | -1.99(-2.69--1.29) |
| Kenya | 1754.31(1165.71-2488.37) | 8.84(5.91-12.46) | -1.07(-1.21--0.92) | 19220.89(15819.89-23161.16) | 94.95(78.32-114.33) | -0.87(-0.90--0.83) | 34135.18(24707.68-46049.09) | 169.58(122.91-228.8) | -0.70(-0.80--0.60) | 527.14(371.49-723.77) | 2.67(1.88-3.66) | -0.66(-0.77--0.56) |
| Kiribati | 26.94(22.02-32.55) | 55.94(45.78-67.52) | -0.64(-0.72--0.57) | 149.24(139.62-159.23) | 310.63(290.80-331.17) | -0.61(-0.63--0.58) | 511.90(342.34-732.25) | 1057.31(706.65-1513.19) | -0.01(-0.06-0.05) | 8.23(5.38-11.98) | 17.08(11.15-24.88) | 0.03(-0.03-0.08) |
| Kuwait | 125.32(82.12-183.01) | 5.37(3.41-8.06) | -0.70(-0.78--0.61) | 2250.64(2036.55-2463.61) | 95.76(86.17-105.25) | -0.46(-0.50--0.42) | 1585.23(1268.16-1974.02) | 68.56(54.82-85.26) | -1.80(-2.88--0.71) | 21.75(16.91-27.60) | 0.91(0.70-1.15) | -2.00(-3.19--0.78) |
| Kyrgyzstan | 203.08(151.72-273.10) | 7.47(5.58-10.08) | -2.90(-2.94--2.85) | 1889.89(1669.20-2135.77) | 69.05(60.97-78.03) | -1.90(-1.96--1.84) | 3302.39(2652.74-4093.09) | 120.79(97.25-149.49) | -3.22(-4.32--2.11) | 52.20(41.34-65.29) | 1.92(1.52-2.39) | -3.27(-4.55--1.97) |
| Lao People's Democratic Republic | 487.55(375.66-632.97) | 15.58(12.02-20.21) | -0.93(-1.02--0.83) | 4375.19(4017.43-4753.62) | 138.31(127.09-150.19) | -0.43(-0.45--0.40) | 15125.89(10161.16-22176.70) | 481.81(323.87-705.85) | -1.68(-1.77--1.60) | 244.31(159.54-362.29) | 7.82(5.11-11.58) | -1.76(-1.84--1.67) |
| Latvia | 19.44(12.02-29.95) | 3.24(1.93-5.14) | -2.56(-2.63--2.49) | 259.17(218.06-304.63) | 44.26(37.00-52.27) | -0.91(-0.95--0.87) | 351.17(269.93-444.29) | 54.95(42.38-69.36) | -2.77(-4.83--0.67) | 5.41(4.01-7.05) | 0.82(0.61-1.07) | -3.12(-5.93--0.23) |
| Lebanon | 150.37(102.16-211.77) | 6.32(4.24-8.97) | -1.81(-1.87--1.75) | 2564.48(2319.43-2821.24) | 105.41(95.18-116.20) | -0.86(-0.89--0.83) | 2682.10(1962.12-3561.80) | 111.89(81.77-148.87) | -3.77(-4.16--3.37) | 38.87(27.21-53.52) | 1.60(1.12-2.21) | -4.14(-4.58--3.70) |
| Lesotho | 85.61(65.55-110.72) | 11.20(8.6-14.46) | 1.63(1.49-1.77) | 656.03(580.62-738.4) | 83.66(74.40-93.78) | 0.67(0.64-0.70) | 2659.99(1648.08-3989.89) | 342.03(211.71-513.25) | 2.51(2.08-2.94) | 43.36(26.16-65.57) | 5.66(3.41-8.56) | 2.62(2.16-3.07) |
| Liberia | 245.69(176.54-332.27) | 11.64(8.43-15.64) | -1.12(-1.32--0.93) | 3149.68(2898.78-3415.55) | 146.75(135.28-158.95) | -1.20(-1.25--1.15) | 6777.62(4195.25-10261.58) | 318.06(197.73-479.72) | -0.53(-1.00--0.05) | 106.26(64.24-163.23) | 5.05(3.07-7.74) | -0.49(-0.98-0.00) |
| Libya | 218.59(157.14-295.29) | 7.10(5.07-9.63) | -1.06(-1.17--0.95) | 3272.71(2991.70-3580.22) | 106.11(96.87-116.20) | -1.16(-1.2-0-1.11) | 6757.72(4368.37-9927.08) | 219.11(141.54-322.10) | -0.71(-1.44-0.03) | 105.5(65.78-158.13) | 3.38(2.11-5.07) | -0.68(-1.47-0.12) |
| Lithuania | 34.46(23.17-50.08) | 3.96(2.61-5.86) | -1.49(-1.80--1.19) | 368.45(310.38-430.20) | 43.16(36.19-50.57) | -0.67(-0.72--0.63) | 334.88(274.69-411.26) | 37.27(30.55-45.79) | -3.04(-4.92--1.13) | 4.84(3.90-6.01) | 0.53(0.43-0.66) | -3.41(-5.35--1.43) |
| Luxembourg | 5.41(2.90-8.94) | 2.34(1.23-3.94) | -3.13(-3.26--3.00) | 128.51(107.43-152.42) | 54.89(45.62-65.29) | -1.44(-1.48--1.41) | 48.56(39.76-58.23) | 20.29(16.62-24.37) | -5.97(-6.78--5.17) | 0.55(0.44-0.67) | 0.22(0.18-0.27) | -7.02(-8.07--5.96) |
| Madagascar | 2339.54(1788.09-3020.85) | 22.36(17.15-28.8) | -1.71(-1.79--1.63) | 17581.14(16120.59-19130.29) | 166.04(152.69-180.10) | -1.34(-1.37--1.31) | 74462.09(48364.59-105484.77) | 688.46(447.31-974.07) | -1.19(-1.29--1.09) | 1189.31(760.19-1702.31) | 11.22(7.18-16.05) | -1.18(-1.28--1.07) |
| Malawi | 728.02(548.45-961.34) | 9.84(7.47-12.92) | -1.25(-1.31--1.20) | 7279.98(6556-8084.68) | 97.86(88.64-108.05) | -0.90(-0.93--0.86) | 23717.29(15415.94-33588.72) | 321.75(210.31-455.68) | -0.38(-0.66--0.10) | 375.09(240.56-537.72) | 5.23(3.37-7.50) | -0.32(-0.59--0.05) |
| Malaysia | 1741.47(1257.93-2335.76) | 12.28(8.85-16.51) | -1.36(-1.42--1.30) | 21286.07(19470.41-23110.19) | 150.11(137.24-163.09) | -1.02(-1.06--0.97) | 30446.67(23249.71-39585.01) | 213.85(163.25-278.15) | -1.62(-1.95--1.29) | 460.37(341.50-610.60) | 3.22(2.39-4.28) | -1.62(-1.99--1.24) |
| Maldives | 31.18(22.53-42.03) | 10.26(7.33-14.01) | -3.20(-3.36--3.03) | 384.81(352.95-418.58) | 129.36(118.35-141.15) | -2.12(-2.20--2.05) | 593.85(425.10-798.15) | 189.11(135.58-254.24) | -4.53(-5.04--4.02) | 9.25(6.28-12.87) | 2.85(1.94-3.98) | -4.78(-5.32--4.24) |
| Mali | 800.11(569.26-1106.63) | 10.26(7.42-14.02) | -1.31(-1.37--1.25) | 11666.33(10737.72-12650.79) | 142.05(131.12-153.62) | -0.78(-0.8--0.76) | 24739.13(16360.67-35219.56) | 308.58(205.82-437.12) | -1.29(-1.57--1.02) | 379.72(244.25-549.36) | 4.88(3.17-7.03) | -1.35(-1.63--1.06) |
| Malta | 5.02(2.96-7.87) | 3.54(2.03-5.66) | -1.61(-1.68--1.54) | 96.37(84.45-109.63) | 66.99(58.41-76.43) | -0.96(-0.98--0.94) | 48.38(40.67-57.03) | 32.71(27.51-38.58) | -3.46(-3.96--2.95) | 0.62(0.51-0.75) | 0.41(0.34-0.49) | -4.00(-4.66--3.34) |
| Marshall Islands | 7.89(6.24-9.91) | 33.99(26.89-42.64) | 0.20(0.08-0.32) | 47.61(44.1-51.27) | 204.52(189.58-220.2) | -0.02(-0.06-0.02) | 235.27(157.51-340.36) | 1013.25(678.50-1464.86) | 0.13(0.04-0.22) | 3.89(2.56-5.69) | 16.78(11.06-24.53) | 0.19(0.12-0.27) |
| Mauritania | 138.49(94.76-196.60) | 8.88(6.16-12.47) | -2.13(-2.20--2.06) | 2193.99(2000.04-2395.79) | 136.40(124.66-148.58) | -1.33(-1.36--1.30) | 2917.47(1812.61-4602.39) | 187.26(115.02-297.82) | -2.50(-2.70--2.29) | 43.95(25.53-71.75) | 2.89(1.66-4.75) | -2.57(-2.81--2.33) |
| Mauritius | 44.82(32.77-60.13) | 9.62(7.01-12.96) | -1.70(-1.79--1.60) | 512.38(465.80-560.10) | 110.53(100.41-120.92) | -1.13(-1.17--1.09) | 1269.34(1073.42-1478.58) | 270.98(229.14-315.60) | -0.20(-1.34-0.95) | 20.60(17.35-24.14) | 4.38(3.69-5.13) | -0.17(-1.42-1.09) |
| Mexico | 2141.99(1311.12-3266.31) | 4.15(2.54-6.33) | -1.62(-1.68--1.57) | 44781.15(37248.69-53224.70) | 86.84(72.23-103.23) | -1.11(-1.15--1.07) | 38369.63(34487.48-42588.78) | 74.33(66.81-82.50) | -1.54(-2.33--0.75) | 590.57(526.82-656.36) | 1.14(1.02-1.27) | -1.54(-2.43--0.64) |
| Micronesia (Federated States of) | 12.52(9.52-16.35) | 31.79(24.2-41.46) | -0.57(-0.62--0.52) | 84.71(78.01-91.48) | 215.62(198.95-232.38) | -0.23(-0.25--0.20) | 376.78(251.27-540.83) | 960.56(642.35-1375.84) | -0.61(-0.63--0.58) | 6.15(4.02-8.90) | 15.83(10.39-22.88) | -0.64(-0.68--0.59) |
| Monaco | 0.31(0.18-0.49) | 3.24(1.89-5.17) | -1.05(-1.11--0.99) | 5.73(4.94-6.63) | 59.39(51-68.82) | -0.24(-0.26--0.22) | 5.58(3.39-8.48) | 56.38(34.59-85.53) | -1.82(-1.98--1.66) | 0.08(0.05-0.13) | 0.83(0.46-1.34) | -2.00(-2.19--1.81) |
| Mongolia | 288.19(226.69-361.83) | 21.01(16.43-26.52) | -0.58(-0.77--0.39) | 2059.81(1897.71-2234.30) | 152.63(140.32-165.81) | -0.92(-0.95--0.89) | 2889.75(2099.40-3870.09) | 210.01(152.65-281.05) | -1.68(-2.84--0.51) | 44.87(31.48-61.39) | 3.23(2.26-4.41) | -1.73(-2.95--0.49) |
| Montenegro | 18.91(14.22-24.76) | 8.64(6.43-11.41) | -2.03(-2.20--1.85) | 237.82(205.73-272.60) | 110.66(95.42-127.20) | -0.91(-0.98--0.84) | 401.59(305.42-526.34) | 183.09(138.96-240.14) | -2.19(-3.33--1.04) | 6.23(4.63-8.34) | 2.80(2.08-3.76) | -2.29(-3.44--1.13) |
| Morocco | 918.63(624.64-1279.91) | 6.20(4.21-8.66) | -2.19(-2.28--2.11) | 14025.83(12652.75-15542.27) | 94.85(85.53-105.13) | -1.16(-1.20--1.12) | 22703.30(13717.38-36255.20) | 153.00(92.47-244.25) | -2.81(-2.94--2.69) | 351.56(197.42-581.15) | 2.36(1.33-3.90) | -2.91(-3.06--2.76) |
| Mozambique | 1535.38(1161.57-2010.10) | 14.77(11.22-19.20) | 0.29(0.24-0.34) | 13274.42(12077.69-14508.05) | 122.82(112.23-133.72) | 0.10(0.08-0.12) | 42886.47(26182.30-65034.34) | 413.39(252.28-625.25) | 0.97(0.78-1.15) | 689.26(409.37-1059.08) | 6.83(4.07-10.47) | 1.03(0.81-1.25) |
| Myanmar | 3294.85(2571.62-4223.26) | 15.01(11.74-19.20) | -1.13(-1.26--0.99) | 29424.69(26890.26-31898.66) | 133.23(121.82-144.34) | -0.62(-0.64--0.59) | 113235.53(78712.39-157807.45) | 514.64(358.04-716.35) | -1.92(-2.12--1.71) | 1837.41(1258.4-2575.81) | 8.38(5.75-11.74) | -1.98(-2.18--1.78) |
| Namibia | 70.04(49.99-95.25) | 7.05(5.06-9.56) | -1.29(-1.34--1.23) | 743.28(651.32-843.64) | 73.63(64.71-83.36) | -0.73(-0.80--0.66) | 1703.41(906.42-2845.11) | 171.36(91.22-285.98) | -1.00(-1.37--0.63) | 27.16(13.62-46.22) | 2.76(1.39-4.70) | -0.99(-1.39--0.59) |
| Nauru | 1.79(1.42-2.27) | 40.82(32.37-51.54) | -0.44(-0.51--0.37) | 11.86(11.04-12.78) | 269.53(251.34-290.04) | -0.25(-0.30--0.21) | 63.11(42.05-95.52) | 1427.10(953.44-2153.71) | -0.05(-0.17-0.07) | 1.03(0.68-1.57) | 23.45(15.45-35.68) | -0.05(-0.18-0.07) |
| Nepal | 700.21(441.53-1051.94) | 5.56(3.54-8.26) | -1.70(-1.77--1.64) | 8394.92(7264.15-9589.40) | 65.60(56.96-74.69) | -0.82(-0.83--0.80) | 14297.47(8601.97-22120.35) | 114.68(69.25-176.84) | -1.97(-2.11--1.83) | 222.26(126.54-354.13) | 1.81(1.04-2.87) | -2.07(-2.22--1.92) |
| Netherlands | 144.79(73.8-253.34) | 2.70(1.36-4.75) | -1.37(-1.43--1.31) | 3143.70(2683.23-3637.19) | 58.35(49.75-67.57) | -0.55(-0.57--0.54) | 1066.58(879.33-1281.30) | 19.54(16.09-23.49) | -3.40(-3.71--3.09) | 11.36(9.52-13.46) | 0.21(0.17-0.24) | -4.39(-5.22--3.55) |
| New Zealand | 36.57(17.55-66.53) | 2.01(0.96-3.68) | -1.09(-1.27--0.91) | 910.27(737.62-1106.83) | 49.45(40.01-60.20) | -0.11(-0.19--0.03) | 345.17(287.71-413.25) | 18.46(15.36-22.13) | -2.70(-4.07--1.31) | 3.76(3.27-4.31) | 0.20(0.17-0.23) | -3.66(-5.40--1.89) |
| Nicaragua | 102.44(58.48-166.26) | 3.63(2.08-5.87) | -1.96(-2.07--1.84) | 2213.07(1957.48-2471.14) | 78.2(69.19-87.30) | -0.88(-0.93--0.82) | 2000.52(1461.24-2706.43) | 71.54(52.25-96.77) | -2.68(-3.26--2.11) | 30.92(21.67-42.92) | 1.11(0.78-1.54) | -2.82(-3.43--2.21) |
| Niger | 865.17(619.29-1182.59) | 11.47(8.37-15.42) | -1.10(-1.15--1.05) | 11938.63(11033.57-12972.24) | 147.95(137.15-160.34) | -0.95(-0.98--0.93) | 17707.81(10757.41-27024.76) | 228.72(139.98-348.21) | -1.00(-1.20--0.79) | 265.45(153.68-416.26) | 3.56(2.08-5.56) | -1.02(-1.33--0.70) |
| Nigeria | 5793.47(3716.23-8560.91) | 7.23(4.69-10.55) | -1.33(-1.45--1.21) | 98524.24(81840.2-117608.46) | 117.15(97.65-139.55) | -0.85(-0.87--0.82) | 98117.02(70134.85-134001.05) | 122.80(88.21-167.09) | -1.88(-2.07--1.69) | 1431.58(975.76-2023.39) | 1.85(1.27-2.60) | -2.02(-2.21--1.83) |
| Niue | 0.10(0.07-0.13) | 17.79(13.18-23.57) | -1.28(-1.31--1.24) | 1.01(0.93-1.09) | 181.44(166.92-196.88) | -0.47(-0.48--0.45) | 3.16(2.22-4.40) | 568.04(398.40-789.89) | -0.17(-0.62-0.29) | 0.05(0.03-0.07) | 8.9(6.09-12.66) | -0.24(-0.71-0.24) |
| North Macedonia | 54(38.55-73.12) | 6.62(4.65-9.10) | -2.02(-2.13--1.92) | 781.87(701.98-862.22) | 95.50(85.45-105.64) | -1.39(-1.44--1.34) | 779.25(554.87-1071.25) | 92.15(65.56-126.84) | -3.44(-4.09--2.79) | 11.15(7.46-15.79) | 1.28(0.86-1.83) | -3.77(-4.47--3.06) |
| Northern Mariana Islands | 2.05(1.50-2.79) | 12.16(8.9-16.51) | -1.18(-1.54--0.82) | 21.71(19.85-23.65) | 129.93(118.79-141.66) | -0.61(-0.83--0.39) | 45.46(32.55-62.25) | 270.54(193.68-370.50) | -1.64(-2.28--0.99) | 0.72(0.5-1.00) | 4.25(2.96-5.92) | -1.73(-2.50--0.96) |
| Norway | 39.12(18.20-71.47) | 2.16(0.99-3.97) | -1.29(-1.52--1.06) | 847.68(688.03-1028.37) | 46.24(37.44-56.15) | -0.39(-0.44--0.34) | 227.28(187.96-275.19) | 12.16(10.02-14.77) | -3.20(-4.01--2.38) | 2.01(1.85-2.18) | 0.10(0.10-0.11) | -4.56(-5.80--3.31) |
| Oman | 166.35(120.43-227.23) | 7.01(5.00-9.65) | -1.93(-2.05--1.81) | 2869.47(2610.13-3142.52) | 113.01(102.39-124.15) | -1.03(-1.06--1.01) | 2656.21(1836.57-3686.95) | 105.55(73.22-146.25) | -2.80(-3.23--2.37) | 38.68(25.26-56.11) | 1.49(0.98-2.16) | -3.00(-3.48--2.53) |
| Pakistan | 10285.23(6842.66-14707.63) | 11.03(7.35-15.73) | -0.49(-0.65--0.33) | 99322.34(81157.82-120151.65) | 105.36(86.20-127.42) | -0.11(-0.14--0.07) | 197036.61(136437.78-273210.24) | 209.79(144.96-290.58) | 0.36(0.15-0.58) | 3080.00(2062.2-4347.46) | 3.32(2.22-4.67) | 0.36(0.20-0.53) |
| Palau | 1.80(1.40-2.28) | 28.42(22.2-36.20) | -0.18(-0.25--0.10) | 13.16(12.18-14.09) | 209.2(193.32-224.51) | -0.03(-0.05--0.01) | 52.25(36.99-71.47) | 839.65(593.69-1149.66) | 0.19(-0.16-0.54) | 0.86(0.60-1.19) | 13.66(9.50-18.96) | 0.18(-0.22-0.58) |
| Palestine | 92.73(57.62-139.74) | 4.35(2.74-6.49) | -1.46(-1.59--1.33) | 1718.11(1517.79-1925.64) | 81.69(72.36-91.41) | -1.01(-1.04--0.98) | 3078.48(2278.51-4024.88) | 145.65(107.85-190.29) | -2.39(-2.89--1.88) | 45.83(32.97-61.05) | 2.20(1.59-2.93) | -2.48(-3.00--1.96) |
| Panama | 70.87(44.28-107.50) | 4.33(2.71-6.55) | -1.79(-1.82--1.75) | 1365.45(1217.58-1527.44) | 83.17(74.19-93.02) | -1.08(-1.11--1.04) | 1168.67(927.31-1446.62) | 71.83(56.99-88.95) | -2.09(-2.98--1.20) | 18.00(13.98-22.72) | 1.11(0.86-1.40) | -2.14(-3.14--1.14) |
| Papua New Guinea | 261.45(180.14-372.27) | 6.41(4.46-9.07) | -0.73(-0.76--0.70) | 3407.57(2987.58-3854.20) | 81.79(71.85-92.36) | -0.39(-0.43--0.34) | 16567.79(9708.28-25447.93) | 405.79(238.58-621.93) | -0.67(-0.84--0.51) | 272.15(154.91-422.95) | 6.72(3.84-10.42) | -0.71(-0.88--0.55) |
| Paraguay | 150.31(99.46-220.19) | 4.96(3.29-7.26) | -2.09(-2.15--2.02) | 2194.48(1911.94-2494.21) | 72.05(62.80-81.87) | -1.18(-1.21--1.15) | 2818.19(1981.86-3862.50) | 93.23(65.58-127.81) | -2.46(-3.42--1.49) | 45.18(31.02-63.05) | 1.50(1.03-2.09) | -2.52(-3.50--1.52) |
| Peru | 924.03(645.4-1269.1) | 6.20(4.32-8.53) | -2.10(-2.18--2.02) | 12968.24(11639.05-14363.18) | 86.69(77.79-96.04) | -1.45(-1.50--1.39) | 21088.80(14634.52-29577.42) | 141.42(98.12-198.33) | -2.15(-3.74--0.54) | 330.52(224.41-471.73) | 2.22(1.50-3.16) | -2.18(-3.86--0.47) |
| Philippines | 6658.13(4571.19-9321.68) | 14.75(10.14-20.62) | 2.48(2.20-2.76) | 57257.44(46944.84-69035.92) | 125.12(102.60-150.81) | 0.84(0.73-0.95) | 174291.89(147290.16-206875.65) | 386.53(326.92-458.11) | 0.09(-0.30-0.47) | 2822.84(2364.13-3369.59) | 6.31(5.29-7.52) | 0.04(-0.36-0.45) |
| Poland | 463.57(282.26-699) | 3.37(1.99-5.22) | -2.79(-2.88--2.71) | 7206.17(5972.89-8632.18) | 54.65(44.97-65.74) | -1.60(-1.63--1.57) | 8462.00(7630.99-9358.12) | 57.52(51.70-63.82) | -3.67(-4.50--2.84) | 127.67(115.58-139.75) | 0.83(0.76-0.91) | -3.95(-4.99--2.90) |
| Portugal | 77.04(43.72-126.77) | 2.46(1.37-4.13) | -4.15(-4.24--4.06) | 1742.50(1431.69-2089.22) | 56.80(46.5-68.22) | -1.68(-1.73--1.63) | 1433.56(1224.25-1656.81) | 45.38(38.84-52.45) | -5.12(-5.90--4.33) | 20.90(17.5-24.43) | 0.65(0.54-0.76) | -5.46(-6.26--4.65) |
| Puerto Rico | 40.49(24.08-63.28) | 3.86(2.28-6.07) | -1.58(-1.71--1.45) | 894.62(797.13-1000.43) | 85.59(76.23-95.75) | -0.55(-0.57--0.52) | 408.02(327.43-501.68) | 38.36(30.79-47.15) | -2.54(-3.99--1.07) | 5.77(4.52-7.24) | 0.54(0.42-0.68) | -2.86(-4.55--1.13) |
| Qatar | 119.31(80.12-170.13) | 6.60(4.31-9.63) | -2.36(-2.46--2.26) | 2318.84(2122.54-2522.57) | 123.92(112.74-135.44) | -1.53(-1.56--1.49) | 1347.48(942.34-1908.45) | 77.21(53.88-109.31) | -3.47(-4.06--2.88) | 17.83(11.12-27.37) | 0.98(0.62-1.50) | -3.80(-4.41--3.18) |
| Republic of Korea | 1001.57(619.06-1539.07) | 5.90(3.57-9.21) | -3.82(-3.97--3.68) | 19099.54(17299.92-21032.72) | 112.8(101.89-124.47) | -2.52(-2.62--2.42) | 10588.34(8210.46-13673.89) | 59.92(46.44-77.26) | -5.03(-5.33--4.73) | 132.42(98.24-180.97) | 0.72(0.54-0.99) | -5.69(-6.05--5.33) |
| Republic of Moldova | 77.07(55.39-105.07) | 5.18(3.59-7.33) | -2.42(-2.77--2.06) | 788.75(683.64-898.48) | 56.10(48.15-64.44) | -1.12(-1.14--1.10) | 1496.05(1234.80-1791.02) | 98.23(81.14-117.58) | -2.74(-4.65--0.79) | 24.12(19.47-29.25) | 1.54(1.24-1.86) | -2.78(-3.53--2.03) |
| Romania | 275.41(194.46-385.63) | 4.75(3.28-6.79) | -2.18(-2.23--2.13) | 4211.19(3701.30-4756.08) | 74.62(65.33-84.53) | -0.78(-0.81--0.75) | 5783.62(4736.40-6992.79) | 97.18(79.53-117.4) | -2.26(-2.65--1.86) | 88.46(71.17-109.06) | 1.45(1.17-1.79) | -2.43(-2.89--1.97) |
| Russian Federation | 4228.24(2896.65-5880.15) | 7.33(4.95-10.35) | -0.35(-0.63--0.06) | 31030.08(25338.37-37736.45) | 57.55(46.77-70.12) | -0.45(-0.50--0.39) | 87905.54(79809.34-94757.82) | 150.26(136.05-162.21) | 0.10(-1.25-1.46) | 1481.01(1342.04-1596.82) | 2.47(2.23-2.67) | 0.26(-1.22-1.77) |
| Rwanda | 526.59(379.01-720.08) | 9.86(7.13-13.45) | -3.93(-4.04--3.82) | 5203.38(4657.20-5761.72) | 96.47(86.57-106.56) | -2.56(-2.65--2.48) | 12182.78(7496.82-18579.13) | 226.36(139.11-344.96) | -4.46(-4.81--4.11) | 192.83(115.65-300.54) | 3.63(2.17-5.65) | -4.51(-4.85--4.17) |
| Saint Kitts and Nevis | 1.53(1.04-2.14) | 6.48(4.40-9.15) | -3.87(-4.18--3.56) | 23.56(21.16-26.13) | 100.30(89.9-111.41) | -1.48(-1.53--1.42) | 20.74(14.53-28.80) | 86.24(60.85-119.31) | -5.78(-6.73--4.82) | 0.33(0.22-0.47) | 1.34(0.90-1.91) | -6.04(-7.06--5.02) |
| Saint Lucia | 4.07(2.87-5.62) | 5.94(4.15-8.27) | -2.11(-2.17--2.05) | 64.87(58.18-72.30) | 95.89(85.91-107) | -0.85(-0.89--0.81) | 73.85(58.94-91.49) | 106.72(85.14-132.23) | -2.86(-3.41--2.30) | 1.18(0.93-1.48) | 1.69(1.33-2.13) | -3.04(-3.61--2.46) |
| Saint Vincent and the Grenadines | 2.76(2.05-3.70) | 6.63(4.91-8.90) | -1.63(-1.68--1.57) | 37.20(33.3-41.49) | 89.68(80.25-100.06) | -0.81(-0.84--0.78) | 63.11(51.45-76.62) | 150.99(123.00-183.44) | -2.22(-3.05--1.39) | 1.04(0.84-1.28) | 2.49(2.01-3.05) | -2.24(-3.13--1.34) |
| Samoa | 17.36(13.13-22.69) | 23.39(17.76-30.42) | 0.02(-0.08-0.13) | 143.23(131.47-155.98) | 191.73(176.39-208.42) | 0.04(0.01-0.08) | 419.96(281.81-606.71) | 566.27(379.87-817.90) | 0.02(-0.03-0.07) | 6.67(4.29-9.86) | 9.1(5.85-13.46) | 0.02(-0.03-0.08) |
| San Marino | 0.25(0.13-0.42) | 2.75(1.43-4.64) | -0.48(-0.55--0.41) | 5.08(4.30-5.92) | 55.79(47.13-65.07) | -0.07(-0.12--0.02) | 2.25(1.51-3.15) | 24.41(16.45-34.14) | -2.79(-3.00--2.59) | 0.03(0.01-0.04) | 0.29(0.16-0.46) | -3.54(-3.79--3.29) |
| Sao Tome and Principe | 13.64(9.90-18.30) | 15.85(11.55-21.23) | -0.57(-0.64--0.50) | 167.40(154.40-182.34) | 193.08(178.29-210.11) | -0.62(-0.66--0.59) | 238.25(144.81-378.82) | 275.16(166.83-438.86) | -0.83(-1.27--0.39) | 3.62(2.05-5.97) | 4.23(2.39-7.00) | -0.93(-1.38--0.47) |
| Saudi Arabia | 1500.48(1130.68-1977.47) | 7.19(5.27-9.68) | -0.77(-0.89--0.66) | 19970.82(18093.6-21954.88) | 99.94(90.21-110.22) | -0.25(-0.30--0.20) | 47438.28(31168.73-70297.94) | 224.34(148.20-331.11) | -1.40(-1.56--1.23) | 778.59(495.01-1178.35) | 3.60(2.29-5.43) | -1.39(-1.57--1.22) |
| Senegal | 628.79(440.99-866.10) | 10.61(7.50-14.51) | -1.04(-1.12--0.95) | 9572.52(8828.18-10364.93) | 156.82(144.90-169.49) | -0.72(-0.74--0.70) | 15167.10(10400.15-21659.65) | 254.42(174.96-362.44) | -1.51(-1.68--1.34) | 231.02(152.85-337.08) | 3.96(2.64-5.77) | -1.55(-1.73--1.37) |
| Serbia | 123.04(79.54-181.53) | 3.94(2.49-5.92) | -2.89(-2.95--2.83) | 2252.16(1976.97-2554.58) | 72.98(63.90-82.92) | -1.40(-1.44--1.37) | 1941.84(1420.01-2562.24) | 59.48(43.53-78.50) | -4.19(-4.53--3.86) | 27.24(18.70-37.47) | 0.81(0.56-1.12) | -4.64(-5.02--4.25) |
| Seychelles | 4.67(3.44-6.31) | 11.43(8.34-15.51) | -1.46(-1.64--1.28) | 49.80(45.59-54.4) | 123.30(112.65-134.95) | -0.66(-0.68--0.64) | 78.93(60.06-101.50) | 188.44(143.34-242.65) | -1.90(-2.15--1.64) | 1.23(0.92-1.61) | 2.90(2.16-3.80) | -2.08(-2.36--1.81) |
| Sierra Leone | 460.92(333.48-626.51) | 13.63(9.92-18.41) | -0.26(-0.32--0.20) | 6565.41(6116.78-7065.44) | 185.93(173.48-199.76) | -0.45(-0.48--0.42) | 12022.10(7504.96-17769.25) | 348.51(218.66-512.07) | -0.27(-0.53--0.01) | 185.22(111.87-280.13) | 5.49(3.34-8.25) | -0.27(-0.54-0.00) |
| Singapore | 92.22(51.52-153.02) | 4.27(2.32-7.27) | -2.42(-2.56--2.27) | 1860.59(1654.76-2073.34) | 86.52(76.53-96.92) | -1.26(-1.30--1.21) | 945.75(781.29-1130.81) | 40.22(32.98-48.37) | -3.12(-4.07--2.16) | 11.61(9.89-13.61) | 0.46(0.39-0.54) | -3.76(-5.13--2.36) |
| Slovakia | 68.48(42.26-103.44) | 3.68(2.20-5.72) | -2.04(-2.11--1.97) | 1197.54(1049.99-1347.40) | 65.24(56.92-73.68) | -0.90(-0.95--0.86) | 1000.65(736.53-1325.21) | 51.01(37.74-67.18) | -3.40(-3.95--2.85) | 13.76(9.55-19.32) | 0.67(0.47-0.94) | -3.89(-4.57--3.21) |
| Slovenia | 16.14(8.58-27.75) | 2.66(1.37-4.68) | -2.64(-2.86--2.41) | 321.54(276.70-370.20) | 52.99(45.42-61.18) | -1.41(-1.45--1.37) | 101.91(76.79-130.12) | 16.12(12.11-20.62) | -5.12(-5.90--4.33) | 0.75(0.57-0.97) | 0.11(0.08-0.14) | -7.33(-8.64--5.99) |
| Solomon Islands | 92.93(73.02-117.49) | 37.30(29.41-47) | 0.15(0.10-0.19) | 546.17(507.20-588.41) | 216.00(200.99-232.31) | 0.02(0.01-0.03) | 1328.16(898.21-1878.42) | 537.01(363.60-758.36) | 0.21(0.00-0.41) | 21.47(13.97-30.79) | 8.76(5.71-12.54) | 0.21(0.02-0.40) |
| Somalia | 1032.13(779.91-1363.35) | 14.20(10.81-18.64) | -1.41(-1.48--1.35) | 8391.50(7607.65-9228.91) | 113.23(103.15-123.94) | -1.00(-1.02--0.98) | 25985.07(14351.88-41343.77) | 347.46(189.79-556) | -1.39(-1.58--1.2) | 409.79(218.04-663.73) | 5.63(2.96-9.16) | -1.41(-1.57--1.24) |
| South Africa | 1831.81(1242.81-2587.31) | 7.19(4.85-10.20) | -2.98(-3.15--2.80) | 17778.02(14478.40-21648.36) | 70.53(57.38-85.91) | -1.84(-1.93--1.74) | 51842.93(44276.84-60763.79) | 202.49(172.71-237.50) | -2.75(-4.00--1.47) | 862.53(734.77-1015.80) | 3.35(2.85-3.94) | -2.78(-4.14--1.40) |
| South Sudan | 280.42(198.83-385.92) | 8.66(6.22-11.80) | -1.84(-1.89--1.78) | 2994.36(2667.76-3348.55) | 91.85(82.34-102.13) | -1.14(-1.17--1.12) | 9106.12(5516.31-14282.94) | 270.30(163.57-425.44) | -0.83(-1.21--0.44) | 142.31(83.5-226.71) | 4.34(2.54-6.94) | -0.83(-1.23--0.42) |
| Spain | 303.06(160.59-512.78) | 2.33(1.21-4.00) | -3.38(-3.52--3.25) | 6604.84(5522.69-7861.58) | 50.64(42.16-60.42) | -2.16(-2.23--2.09) | 3750.25(3188.84-4347.15) | 27.95(23.74-32.45) | -5.42(-6.08--4.75) | 49.78(41.5-58.66) | 0.36(0.30-0.43) | -5.96(-6.74--5.16) |
| Sri Lanka | 521.95(345.07-756.55) | 6.42(4.24-9.30) | -1.60(-1.67--1.54) | 7464.88(6708.03-8286.72) | 92.02(82.66-102.18) | -0.98(-1.00--0.96) | 13310.18(8857.38-18811.85) | 163.42(108.92-230.89) | -2.00(-3.82--0.15) | 202.47(126.46-296.83) | 2.48(1.55-3.63) | -2.07(-4.03--0.07) |
| Sudan | 1438.99(1030.63-1940.92) | 8.28(5.98-11.09) | -2.24(-2.34--2.14) | 18468.67(16676.28-20415.12) | 104.79(94.88-115.54) | -1.25(-1.29--1.21) | 47343.55(26168.39-74887.66) | 272.21(150.05-431.02) | -2.57(-2.67--2.48) | 740.70(391.08-1196.03) | 4.33(2.28-6.99) | -2.65(-2.79--2.50) |
| Suriname | 20.41(15.67-26.29) | 9.42(7.23-12.13) | -1.18(-1.25--1.10) | 224.57(203.07-246.91) | 104.18(94.19-114.58) | -0.93(-0.96--0.89) | 519.16(367.86-715.73) | 239.41(169.64-329.98) | -1.50(-3.31-0.33) | 8.63(6.02-12.00) | 3.97(2.77-5.52) | -1.54(-3.36-0.31) |
| Sweden | 92.98(46.86-161.68) | 2.77(1.38-4.88) | -0.38(-0.49--0.27) | 2343.05(1898.57-2860.91) | 69.60(56.22-85.20) | 0.16(0.10-0.23) | 601.15(474.85-758.51) | 17.63(13.90-22.27) | -2.98(-3.31--2.65) | 5.12(4.20-6.20) | 0.15(0.12-0.18) | -4.51(-5.15--3.87) |
| Switzerland | 58.30(26.33-107.36) | 2.00(0.88-3.74) | -1.67(-1.90--1.45) | 1313.00(1089.06-1555.89) | 44.62(36.84-52.99) | -0.85(-0.90--0.80) | 380.15(310.68-459.58) | 12.62(10.29-15.29) | -4.98(-5.79--4.16) | 3.57(2.94-4.27) | 0.11(0.09-0.14) | -6.41(-7.56--5.24) |
| Syrian Arab Republic | 665.69(508.45-861.01) | 13.62(10.48-17.39) | -2.25(-2.38--2.12) | 7336.68(6738.49-7951.80) | 159.41(146.79-172.42) | -1.74(-1.80--1.68) | 15488.64(11135.08-21473.30) | 317.5(227.89-444.21) | -2.87(-3.31--2.44) | 232.97(163.29-329.45) | 4.85(3.39-6.93) | -2.93(-3.42--2.44) |
| Taiwan (Province of China) | 719.4(483.94-1009.16) | 8.67(5.70-12.38) | -1.72(-1.78--1.66) | 11142.43(10158.37-12212.66) | 138.67(126.01-152.46) | -1.07(-1.10--1.04) | 9280.22(7786.38-10934.44) | 108.77(90.90-128.70) | -2.64(-3.48--1.80) | 127.35(108.63-148.10) | 1.44(1.23-1.68) | -2.99(-4.02--1.95) |
| Tajikistan | 262.82(182.26-374.32) | 6.40(4.44-9.12) | -2.04(-2.13--1.95) | 2805.37(2436.33-3200.45) | 67.66(58.80-77.13) | -1.12(-1.15--1.09) | 5984.77(4006.10-8542.88) | 144.86(96.98-206.86) | -2.19(-2.84--1.53) | 93.44(60.16-136.56) | 2.27(1.46-3.32) | -2.29(-2.95--1.63) |
| Thailand | 2881.93(2233.44-3693.55) | 12.69(9.78-16.32) | -0.61(-0.67--0.56) | 26254.46(24172.57-28532.88) | 117.41(107.87-127.83) | -0.83(-0.89--0.78) | 77547.42(54288.63-106859.74) | 337.26(235.28-465.27) | 1.02(0.08-1.97) | 1276.12(874.49-1785.30) | 5.48(3.74-7.68) | 1.25(0.24-2.27) |
| Timor-Leste | 54.37(38.57-75.36) | 11.12(7.99-15.27) | -0.04(-0.21-0.14) | 570.31(514.25-627.85) | 111.43(100.95-122.23) | -0.19(-0.22--0.17) | 1814.68(1063.98-2697.93) | 366.92(210.87-545.13) | -0.40(-1.04-0.25) | 28.27(15.85-42.91) | 5.90(3.25-8.94) | -0.44(-1.08-0.21) |
| Togo | 408.38(298.46-552.30) | 12.91(9.49-17.41) | -0.82(-1.03--0.61) | 5059.79(4684.18-5469.68) | 156.43(145.01-168.89) | -0.70(-0.72--0.68) | 9865.66(6137.13-14516.63) | 310.54(193.83-455.74) | -1.01(-1.29--0.72) | 154.83(92.91-231.98) | 4.94(2.98-7.38) | -1.02(-1.32--0.72) |
| Tokelau | 0.08(0.06-0.10) | 16.04(11.84-21.33) | -1.01(-1.06--0.95) | 0.71(0.64-0.77) | 147.27(134.38-160.55) | -0.36(-0.40--0.32) | 3.09(2.14-4.16) | 640.94(445.46-861.25) | 0.16(-0.26-0.58) | 0.05(0.03-0.07) | 10.23(6.96-13.89) | 0.09(-0.33-0.50) |
| Tonga | 3.64(2.58-5.03) | 10.02(7.16-13.74) | -0.50(-0.56--0.43) | 43.29(39.16-47.55) | 118.60(107.54-129.92) | -0.17(-0.20--0.14) | 80.42(54.00-119.13) | 224.49(150.89-332.66) | -0.10(-0.27-0.08) | 1.22(0.78-1.85) | 3.45(2.22-5.25) | -0.10(-0.25-0.06) |
| Trinidad and Tobago | 26.01(17.49-37.24) | 4.89(3.24-7.10) | -1.87(-1.94--1.80) | 476.51(427.2-528.77) | 91.50(81.83-101.76) | -0.82(-0.85--0.79) | 698.36(516.54-918.63) | 123.50(91.66-161.91) | -1.29(-2.42--0.16) | 11.63(8.40-15.53) | 2.01(1.46-2.68) | -1.26(-2.50--0.01) |
| Tunisia | 255.36(173.94-365.64) | 5.63(3.78-8.14) | -1.19(-1.22--1.17) | 4014.38(3622.13-4448.24) | 88.50(79.66-98.22) | -0.61(-0.64--0.59) | 5166.10(3311.85-7628.38) | 111.47(71.15-165.02) | -1.85(-1.98--1.71) | 78.92(47.29-120.76) | 1.67(1.00-2.57) | -1.96(-2.11--1.81) |
| Turkey | 1893.70(1221.54-2789.06) | 5.84(3.74-8.64) | -2.75(-2.81--2.69) | 33248.88(30071.76-36542.88) | 102.31(92.44-112.54) | -1.88(-1.95--1.82) | 31123.37(22537.23-41796.25) | 95.58(69.10-128.5) | -3.84(-4.47--3.21) | 443.71(303.49-613.47) | 1.35(0.92-1.87) | -4.11(-4.8--3.42) |
| Turkmenistan | 309.2(241.71-398.27) | 14.88(11.63-19.18) | 0.64(0.56-0.73) | 2469.61(2249.16-2706.37) | 118.55(107.97-129.92) | 0.44(0.40-0.48) | 6849.54(5278.57-8829) | 329.51(253.95-424.85) | 1.38(0.17-2.61) | 109.04(82.29-142.54) | 5.25(3.96-6.87) | 1.43(-0.01-2.89) |
| Tuvalu | 1.27(0.99-1.64) | 27.11(21.01-34.71) | -0.46(-0.58--0.35) | 8.53(7.86-9.23) | 180.57(166.63-195.08) | -0.36(-0.39--0.33) | 36.08(25.57-49.53) | 762.67(540.09-1045.88) | -0.94(-1.02--0.85) | 0.59(0.41-0.81) | 12.50(8.73-17.34) | -0.97(-1.05--0.88) |
| Uganda | 1469.46(1083.84-1966.43) | 9.56(7.12-12.70) | -0.94(-1.12--0.75) | 15277.90(13666.93-16902.28) | 98.07(88.21-107.92) | -0.70(-0.75--0.66) | 33506.27(21023.74-48763.28) | 217.05(136.04-316.30) | -1.24(-1.52--0.96) | 515.45(311.61-766.96) | 3.45(2.08-5.13) | -1.26(-1.55--0.97) |
| Ukraine | 1708.02(1165.29-2437.30) | 10.12(6.84-14.57) | 0.66(0.37-0.95) | 13309.90(10965.41-16083.17) | 84.40(69.24-102.26) | 0.10(-0.01-0.21) | 24722.83(17822.69-32672.10) | 141.71(102.61-186.42) | 0.28(-1.16-1.74) | 401.98(280.53-541.67) | 2.24(1.57-3.01) | 0.39(-1.15-1.96) |
| United Arab Emirates | 393.73(280.79-546.43) | 6.85(4.66-9.82) | -1.20(-1.31--1.09) | 5273.03(4818.02-5777.74) | 108.56(98.36-119.81) | -1.26(-1.29--1.24) | 3875.81(2555.65-5475.31) | 103.31(70.38-144.18) | -1.53(-3.86-0.86) | 55.15(32.81-82.66) | 1.41(0.88-2.06) | -1.87(-3.88-0.19) |
| United Kingdom | 596.39(330.59-988.17) | 2.64(1.44-4.44) | -1.11(-1.20--1.02) | 11709.52(9667.11-14082.07) | 51.74(42.58-62.27) | -0.82(-0.86--0.78) | 5664.50(5113.23-6300.10) | 24.24(21.81-27.05) | -2.38(-3.21--1.54) | 72.13(69.06-75.17) | 0.30(0.29-0.32) | -2.74(-3.78--1.68) |
| United Republic of Tanzania | 2107.75(1533.38-2849.64) | 9.83(7.22-13.18) | -1.30(-1.33--1.27) | 22934.22(20665.72-25237.33) | 105.65(95.51-115.95) | -0.64(-0.67--0.61) | 43525.59(27945.06-63473.57) | 201.61(130.20-293.56) | -1.62(-1.82--1.41) | 668.99(416.05-995.07) | 3.17(1.98-4.70) | -1.69(-1.90--1.48) |
| United States Virgin Islands | 1.00(0.61-1.55) | 4.14(2.48-6.52) | -1.54(-1.85--1.22) | 19.63(17.48-21.84) | 82.93(73.76-92.39) | -0.42(-0.46--0.38) | 26.00(15.29-41.43) | 107.91(63.22-173.08) | -0.59(-1.52-0.35) | 0.42(0.23-0.68) | 1.71(0.95-2.79) | -0.70(-1.68-0.28) |
| United States of America | 3608.89(2074.08-5710.05) | 3.16(1.80-5.04) | -0.76(-0.95--0.57) | 90343.56(73334.8-110118.65) | 79.42(64.34-96.93) | -0.31(-0.41--0.21) | 55465.54(50061.64-61820.23) | 47.72(43.00-53.28) | -0.93(-1.34--0.52) | 745.81(686.91-801.92) | 0.63(0.58-0.68) | -1.12(-1.70--0.54) |
| Uruguay | 64.03(39.37-98.98) | 5.31(3.25-8.22) | -2.22(-2.28--2.16) | 1164.70(1042.23-1288.06) | 96.30(86.14-106.57) | -1.15(-1.18--1.12) | 887.35(761.46-1035.59) | 72.83(62.48-85.00) | -3.22(-3.46--2.97) | 12.39(10.57-14.49) | 1.01(0.86-1.18) | -3.54(-3.83--3.25) |
| Uzbekistan | 1381.84(1039.85-1791.59) | 9.57(7.14-12.51) | -0.76(-0.92--0.61) | 12263.45(10918.44-13800.34) | 85.52(75.95-96.50) | -0.23(-0.24--0.22) | 19760.39(16444.18-23307.45) | 137.03(113.85-161.90) | -2.88(-4.63--1.09) | 302.91(249.13-362.06) | 2.09(1.71-2.50) | -3.08(-5.00--1.13) |
| Vanuatu | 37.64(29.38-47.62) | 32.61(25.54-41.16) | 0.36(0.27-0.44) | 260.57(241.92-280.90) | 224.20(208.52-241.34) | 0.24(0.22-0.26) | 992.06(650.04-1395.00) | 853.11(560.39-1198.23) | -0.03(-0.20-0.14) | 15.98(10.19-22.85) | 13.90(8.88-19.84) | -0.05(-0.23-0.13) |
| Venezuela (Bolivarian Republic of) | 614.39(434.42-845.12) | 6.30(4.42-8.71) | -1.29(-1.42--1.16) | 9602.07(8648.02-10639.73) | 99.97(89.86-110.89) | -0.63(-0.70--0.56) | 12898.91(9315.08-16804.29) | 130.17(94.05-169.51) | -1.02(-1.61--0.43) | 209.15(146.47-277.60) | 2.08(1.46-2.76) | -1.06(-1.59--0.53) |
| Viet Nam | 4566.11(3383.32-6083.08) | 10.91(8-14.69) | -0.97(-1.12--0.82) | 46871.78(42761.75-51106.01) | 115.09(104.76-125.79) | -0.31(-0.34--0.28) | 103186.86(70996.65-149594.64) | 243.68(167.82-352.66) | -1.42(-1.54--1.29) | 1667.73(1110.60-2488.87) | 3.88(2.58-5.78) | -1.54(-1.68--1.40) |
| Yemen | 1090.75(801.28-1459.26) | 8.37(6.21-11.10) | -1.69(-1.71--1.67) | 12198.96(10886.48-13541.10) | 92.15(82.43-102.04) | -1.02(-1.06--0.98) | 30511.98(17680.15-47310.79) | 236.66(138.17-365.54) | -1.64(-2.09--1.18) | 491.91(276.73-771.80) | 3.85(2.18-6.02) | -1.72(-2.18--1.25) |
| Zambia | 839.33(638.73-1098.84) | 11.43(8.77-14.88) | -1.39(-1.47--1.32) | 7841.92(7056.98-8652.14) | 104.82(94.72-115.26) | -0.92(-0.95--0.89) | 22242.05(13610.10-33946.69) | 302.57(186.08-461.35) | -1.31(-1.47--1.15) | 354.32(211.3-545.98) | 4.92(2.96-7.57) | -1.31(-1.48--1.15) |
| Zimbabwe | 360.69(257.78-491.91) | 6.04(4.34-8.19) | 0.57(0.40-0.75) | 3845.11(3327.32-4408.84) | 64.17(55.77-73.34) | 0.06(-0.01-0.13) | 16973.11(10818.65-25711.61) | 279.07(177.49-424.05) | 3.16(1.94-4.39) | 269.37(168.54-413.91) | 4.50(2.81-6.95) | 3.36(2.11-4.62) |

Abbreviations: ASR, Age-Standardized Rate; AAPC, Average Annual Percentage Change; DALY, Disability-Adjusted Life Year; CI, Confidence Interval; UI, Uncertainty Interval. *Numbers in parentheses represent 95% uncertainty intervals (UI).

**Table S3. Joinpoint Analysis of Age-Standardized Rates of AYA ICH from 1990 to 2021. (A-D)**

1. **Joinpoint Analysis of Age-Standardized Incidence Rates of AYA ICH from 1990 to 2021**

| **sex** | **start Obs** | **End Obs** | **measure** | **val** | **lower** | **upper** | **P val** |
| --- | --- | --- | --- | --- | --- | --- | --- |
| Both | 1990 | 2021 | AAPC | -1.24 | -1.31 | -1.16 | 0.000 |
| Female | 1990 | 2021 | AAPC | -1.68 | -1.75 | -1.61 | 0.000 |
| Male | 1990 | 2021 | AAPC | -0.91 | -0.97 | -0.86 | 0.000 |
| Both | 1990 | 1997 | APC | -0.32 | -0.43 | -0.21 | 0.000 |
| Both | 1997 | 2006 | APC | -1.05 | -1.14 | -0.96 | 0.000 |
| Both | 2006 | 2014 | APC | -2.91 | -3.02 | -2.81 | 0.000 |
| Both | 2014 | 2019 | APC | 0.04 | -0.21 | 0.30 | 0.719 |
| Both | 2019 | 2021 | APC | -1.68 | -2.48 | -0.88 | 0.000 |
| Female | 1990 | 1995 | APC | -0.28 | -0.41 | -0.14 | 0.001 |
| Female | 1995 | 2001 | APC | -1.33 | -1.46 | -1.19 | 0.000 |
| Female | 2001 | 2005 | APC | -2.12 | -2.42 | -1.82 | 0.000 |
| Female | 2005 | 2014 | APC | -3.59 | -3.65 | -3.52 | 0.000 |
| Female | 2014 | 2019 | APC | 0.33 | 0.13 | 0.52 | 0.003 |
| Female | 2019 | 2021 | APC | -1.64 | -2.24 | -1.03 | 0.000 |
| Male | 1990 | 2005 | APC | -0.29 | -0.31 | -0.27 | 0.000 |
| Male | 2005 | 2010 | APC | -1.64 | -1.80 | -1.49 | 0.000 |
| Male | 2010 | 2014 | APC | -2.97 | -3.21 | -2.73 | 0.000 |
| Male | 2014 | 2019 | APC | 0.02 | -0.14 | 0.18 | 0.776 |
| Male | 2019 | 2021 | APC | -1.87 | -2.36 | -1.37 | 0.000 |

1. **Joinpoint Analysis of Age-Standardized Prevalence Rates of AYA ICH from 1990 to 2021**

| **sex** | **start Obs** | **End Obs** | **measure** | **val** | **lower** | **upper** | **P val** |
| --- | --- | --- | --- | --- | --- | --- | --- |
| Both | 1990 | 2021 | AAPC | -0.90 | -0.94 | -0.87 | 0.000 |
| Female | 1990 | 2021 | AAPC | -1.10 | -1.14 | -1.06 | 0.000 |
| Male | 1990 | 2021 | AAPC | -0.71 | -0.75 | -0.68 | 0.000 |
| Both | 1990 | 2000 | APC | -0.68 | -0.71 | -0.65 | 0.000 |
| Both | 2000 | 2005 | APC | -1.23 | -1.35 | -1.11 | 0.000 |
| Both | 2005 | 2015 | APC | -1.61 | -1.65 | -1.58 | 0.001 |
| Both | 2015 | 2019 | APC | -0.38 | -0.58 | -0.19 | 0.000 |
| Both | 2019 | 2021 | APC | 1.34 | 0.95 | 1.73 | 0.000 |
| Female | 1990 | 2000 | APC | -0.83 | -0.86 | -0.80 | 0.000 |
| Female | 2000 | 2006 | APC | -1.55 | -1.64 | -1.47 | 0.000 |
| Female | 2006 | 2014 | APC | -2.01 | -2.06 | -1.96 | 0.000 |
| Female | 2014 | 2019 | APC | -0.67 | -0.80 | -0.54 | 0.000 |
| Female | 2019 | 2021 | APC | 1.58 | 1.17 | 1.98 | 0.000 |
| Male | 1990 | 1999 | APC | -0.51 | -0.54 | -0.47 | 0.000 |
| Male | 1999 | 2005 | APC | -0.88 | -0.95 | -0.80 | 0.000 |
| Male | 2005 | 2015 | APC | -1.33 | -1.36 | -1.30 | 0.000 |
| Male | 2015 | 2019 | APC | -0.37 | -0.54 | -0.19 | 0.000 |
| Male | 2019 | 2021 | APC | 1.29 | 0.93 | 1.64 | 0.000 |

1. **Joinpoint Analysis of Age-Standardized Mortality Rates of AYA ICH from 1990 to 2021**

| **sex** | **start Obs** | **End Obs** | **measure** | **val** | **lower** | **upper** | **P val** |
| --- | --- | --- | --- | --- | --- | --- | --- |
| Both | 1990 | 2021 | AAPC | -1.40 | -1.59 | -1.21 | 0.000 |
| Female | 1990 | 2021 | AAPC | -1.91 | -2.05 | -1.78 | 0.000 |
| Male | 1990 | 2021 | AAPC | -1.06 | -1.29 | -0.82 | 0.000 |
| Both | 1990 | 1994 | APC | -0.66 | -1.08 | -0.24 | 0.005 |
| Both | 1994 | 1997 | APC | -1.66 | -2.97 | -0.34 | 0.017 |
| Both | 1997 | 2003 | APC | -0.08 | -0.38 | 0.22 | 0.561 |
| Both | 2003 | 2009 | APC | -1.91 | -2.20 | -1.62 | 0.000 |
| Both | 2009 | 2012 | APC | -3.12 | -4.40 | -1.81 | 0.000 |
| Both | 2012 | 2021 | APC | -1.58 | -1.70 | -1.46 | 0.000 |
| Female | 1990 | 1997 | APC | -1.31 | -1.51 | -1.11 | 0.000 |
| Female | 1997 | 2003 | APC | -0.82 | -1.15 | -0.48 | 0.000 |
| Female | 2003 | 2012 | APC | -2.96 | -3.12 | -2.80 | 0.000 |
| Female | 2012 | 2019 | APC | -2.29 | -2.54 | -2.04 | 0.000 |
| Female | 2019 | 2021 | APC | -1.20 | -2.68 | 0.31 | 0.112 |
| Male | 1990 | 1994 | APC | -0.35 | -0.87 | 0.17 | 0.170 |
| Male | 1994 | 1997 | APC | -1.71 | -3.31 | -0.07 | 0.042 |
| Male | 1997 | 2003 | APC | 0.40 | 0.03 | 0.77 | 0.036 |
| Male | 2003 | 2009 | APC | -1.24 | -1.60 | -0.88 | 0.000 |
| Male | 2009 | 2012 | APC | -3.15 | -4.74 | -1.54 | 0.001 |
| Male | 2012 | 2021 | APC | -1.28 | -1.43 | -1.13 | 0.000 |

1. **Joinpoint Analysis of Age-Standardized DALYs Rates of AYAs ICH from 1990 to 2021**

| **sex** | **start Obs** | **End Obs** | **measure** | **val** | **lower** | **upper** | **P val** |
| --- | --- | --- | --- | --- | --- | --- | --- |
| Both | 1990 | 2021 | AAPC | -1.37 | -1.59 | -1.15 | 0.000 |
| Female | 1990 | 2021 | AAPC | -1.80 | -1.93 | -1.67 | 0.000 |
| Male | 1990 | 2021 | AAPC | -1.05 | -1.28 | -0.81 | 0.000 |
| Both | 1990 | 1994 | APC | -0.68 | -1.16 | -0.19 | 0.010 |
| Both | 1994 | 1997 | APC | -1.53 | -3.04 | 0.00 | 0.050 |
| Both | 1997 | 2003 | APC | -0.19 | -0.54 | 0.15 | 0.249 |
| Both | 2003 | 2010 | APC | -1.98 | -2.23 | -1.72 | 0.000 |
| Both | 2010 | 2013 | APC | -2.91 | -4.40 | -1.40 | 0.001 |
| Both | 2013 | 2021 | APC | -1.42 | -1.58 | -1.25 | 0.000 |
| Female | 1990 | 2003 | APC | -1.04 | -1.13 | -0.95 | 0.000 |
| Female | 2003 | 2012 | APC | -2.81 | -2.98 | -2.63 | 0.000 |
| Female | 2012 | 2019 | APC | -2.21 | -2.48 | -1.93 | 0.000 |
| Female | 2019 | 2021 | APC | -0.80 | -2.45 | 0.87 | 0.328 |
| Male | 1990 | 1994 | APC | -0.40 | -0.93 | 0.13 | 0.126 |
| Male | 1994 | 1997 | APC | -1.61 | -3.24 | 0.05 | 0.057 |
| Male | 1997 | 2003 | APC | 0.29 | -0.09 | 0.67 | 0.121 |
| Male | 2003 | 2010 | APC | -1.39 | -1.67 | -1.11 | 0.000 |
| Male | 2010 | 2013 | APC | -2.97 | -4.58 | -1.33 | 0.002 |
| Male | 2013 | 2021 | APC | -1.13 | -1.31 | -0.94 | 0.000 |

Note: Joinpoint regression analysis of age-standardized rates for ICH among AYAs from 1990 to 2021. The analysis includes ASIR, ASPR, ASMR, and ASR for DALYs. The table outlines the AAPC and APC during specific periods, highlighting significant trends and shifts in the data. Abbreviations: AAPC: Annual Average Percentage Change; APC: Annual Percentage Change; UI: Uncertainty Interval.

**S4 table. BAPC Prediction of ICH Cases and Rates Among AYAs from 2021 to 2040.**

|  | The cases resulted by BAPC prediction | | | | | | | | | | | | | | | | | | | | | | | |
| --- | --- | --- | --- | --- | --- | --- | --- | --- | --- | --- | --- | --- | --- | --- | --- | --- | --- | --- | --- | --- | --- | --- | --- | --- |
|  | Incidence | | | | | | Prevalence | | | | | | Deaths | | | | | | DALYs | | | | | |
|  | ASR | | | Number | | | ASR | | | Number | | | ASR | | | Number | | | ASR | | | Number | | |
|  | Both | Male | Female | Both | Male | Female | Both | Male | Female | Both | Male | Female | Both | Male | Female | Both | Male | Female | Both | Male | Female | Both | Male | Female |
| 2021 | 8.1 | 9.8 | 6.4 | 246877.9 | 150776.9 | 96106.3 | 94.6 | 97.6 | 91.4 | 2853189.3 | 1493504.1 | 1359390.8 | 2.8 | 3.6 | 2.0 | 84981.6 | 55136.5 | 29815.2 | 177.4 | 221.2 | 132.7 | 5385169.9 | 3404874.6 | 1980237.1 |
| 2022 | 8.0 | 9.7 | 6.4 | 249820.8 | 152769.5 | 97278.0 | 95.5 | 98.5 | 92.2 | 2949495.7 | 1541091.2 | 1406434.9 | 2.8 | 3.6 | 1.9 | 86182.3 | 56437.6 | 29909.6 | 175.6 | 221.2 | 129.9 | 5463708.8 | 3483503.9 | 1989014.2 |
| 2023 | 8.0 | 9.6 | 6.3 | 249124.2 | 152284.0 | 97092.2 | 96.7 | 99.7 | 93.5 | 3005982.2 | 1569373.3 | 1433414.0 | 2.7 | 3.5 | 1.9 | 85713.4 | 56241.5 | 29636.5 | 173.8 | 219.1 | 128.4 | 5441528.9 | 3473475.0 | 1977386.2 |
| 2024 | 7.9 | 9.5 | 6.3 | 248150.7 | 151571.5 | 96844.7 | 98.0 | 100.9 | 94.9 | 3059757.3 | 1596157.1 | 1459135.7 | 2.7 | 3.5 | 1.9 | 85104.9 | 55917.7 | 29349.9 | 171.9 | 216.8 | 127.1 | 5410985.9 | 3456087.7 | 1964741.8 |
| 2025 | 7.8 | 9.4 | 6.2 | 246988.4 | 150692.6 | 96571.0 | 99.3 | 102.1 | 96.2 | 3112313.8 | 1622202.1 | 1484324.3 | 2.7 | 3.5 | 1.9 | 84375.9 | 55480.1 | 29059.1 | 170.0 | 214.3 | 125.8 | 5373337.4 | 3432252.9 | 1951667.9 |
| 2026 | 7.8 | 9.3 | 6.2 | 245834.3 | 149787.7 | 96321.3 | 100.7 | 103.3 | 97.6 | 3164655.1 | 1648045.2 | 1509446.5 | 2.6 | 3.4 | 1.8 | 83552.2 | 54955.3 | 28766.5 | 168.1 | 211.6 | 124.5 | 5330254.8 | 3403605.6 | 1938245.7 |
| 2027 | 7.7 | 9.2 | 6.2 | 244709.0 | 148893.0 | 96103.5 | 102.0 | 104.5 | 99.0 | 3216101.5 | 1673532.8 | 1534086.2 | 2.6 | 3.4 | 1.8 | 82657.8 | 54371.2 | 28472.4 | 166.1 | 208.9 | 123.4 | 5283117.5 | 3371813.7 | 1924361.2 |
| 2028 | 7.6 | 9.1 | 6.1 | 243485.3 | 147961.6 | 95850.2 | 103.3 | 105.7 | 100.3 | 3266140.7 | 1698570.4 | 1557844.2 | 2.5 | 3.3 | 1.8 | 81681.4 | 53719.7 | 28164.4 | 164.1 | 206.0 | 122.2 | 5231462.0 | 3336452.6 | 1909489.1 |
| 2029 | 7.6 | 9.1 | 6.1 | 242150.7 | 146988.2 | 95547.9 | 104.7 | 106.9 | 101.7 | 3315180.1 | 1723333.4 | 1580911.3 | 2.5 | 3.3 | 1.8 | 80633.4 | 53002.4 | 27844.6 | 162.1 | 203.1 | 121.1 | 5175805.2 | 3297508.6 | 1893909.7 |
| 2030 | 7.5 | 9.0 | 6.1 | 240933.6 | 146105.5 | 95282.2 | 106.1 | 108.2 | 103.1 | 3366286.3 | 1749224.9 | 1604873.8 | 2.5 | 3.2 | 1.8 | 79596.5 | 52272.9 | 27539.5 | 160.2 | 200.2 | 120.1 | 5120795.3 | 3257903.3 | 1879248.8 |
| 2031 | 7.5 | 8.9 | 6.0 | 240110.5 | 145475.4 | 95148.5 | 107.5 | 109.5 | 104.6 | 3421901.8 | 1777323.6 | 1631034.0 | 2.4 | 3.2 | 1.7 | 78646.3 | 51583.8 | 27271.0 | 158.3 | 197.2 | 119.1 | 5070744.2 | 3220592.2 | 1866754.4 |
| 2032 | 7.4 | 8.8 | 6.0 | 239851.8 | 145192.7 | 95226.9 | 109.0 | 110.8 | 106.2 | 3483301.1 | 1808277.9 | 1660078.0 | 2.4 | 3.1 | 1.7 | 77839.2 | 50976.9 | 27059.8 | 156.4 | 194.2 | 118.2 | 5028782.4 | 3187966.0 | 1857480.3 |
| 2033 | 7.4 | 8.8 | 6.0 | 240009.9 | 145197.6 | 95449.2 | 110.5 | 112.2 | 107.7 | 3549793.5 | 1841821.2 | 1691607.2 | 2.4 | 3.0 | 1.7 | 77134.8 | 50437.8 | 26886.5 | 154.4 | 191.1 | 117.2 | 4992373.0 | 3159033.6 | 1850123.3 |
| 2034 | 7.3 | 8.7 | 6.0 | 240485.5 | 145428.9 | 95782.7 | 112.0 | 113.6 | 109.3 | 3621448.5 | 1878071.5 | 1725567.7 | 2.3 | 3.0 | 1.7 | 76471.4 | 49924.8 | 26735.3 | 152.3 | 187.9 | 116.2 | 4958622.0 | 3131743.8 | 1844149.2 |
| 2035 | 7.3 | 8.6 | 5.9 | 241245.0 | 145842.7 | 96229.8 | 113.7 | 115.1 | 111.0 | 3698426.8 | 1916919.0 | 1762167.5 | 2.3 | 2.9 | 1.7 | 75821.6 | 49412.8 | 26603.6 | 150.2 | 184.6 | 115.2 | 4925913.8 | 3104652.0 | 1839463.5 |
| 2036 | 7.3 | 8.6 | 5.9 | 242296.9 | 146424.8 | 96800.5 | 115.4 | 116.6 | 112.7 | 3780258.8 | 1957910.9 | 1801337.6 | 2.3 | 2.9 | 1.6 | 75169.8 | 48888.9 | 26486.8 | 148.1 | 181.2 | 114.2 | 4893239.0 | 3077009.6 | 1835693.6 |
| 2037 | 7.2 | 8.5 | 5.9 | 243604.3 | 147130.0 | 97497.9 | 117.1 | 118.2 | 114.5 | 3865211.9 | 2000081.6 | 1842376.0 | 2.2 | 2.8 | 1.6 | 74508.5 | 48347.6 | 26382.1 | 145.8 | 177.7 | 113.1 | 4859944.0 | 3048446.0 | 1832472.7 |
| 2038 | 7.2 | 8.5 | 5.9 | 245161.6 | 147946.3 | 98320.4 | 118.8 | 119.7 | 116.3 | 3953321.1 | 2043384.9 | 1885363.1 | 2.2 | 2.7 | 1.6 | 73853.4 | 47802.9 | 26287.1 | 143.5 | 174.2 | 112.0 | 4827029.3 | 3019693.5 | 1829654.0 |
| 2039 | 7.1 | 8.4 | 5.8 | 247012.5 | 148903.0 | 99280.3 | 120.6 | 121.4 | 118.1 | 4045580.7 | 2088392.9 | 1930724.7 | 2.1 | 2.7 | 1.6 | 73237.1 | 47279.8 | 26207.7 | 141.3 | 170.8 | 110.9 | 4796520.4 | 2992191.0 | 1827735.6 |
| 2040 | 7.1 | 8.4 | 5.8 | 249137.4 | 149988.7 | 100373.2 | 122.5 | 123.1 | 120.0 | 4142187.7 | 2135210.1 | 1978532.4 | 2.1 | 2.6 | 1.6 | 72665.7 | 46783.4 | 26144.9 | 139.1 | 167.4 | 109.8 | 4768727.8 | 2966180.1 | 1826859.4 |

Abbreviations: ASR, Age-Standardized Rate; DALY, Disability-Adjusted Life Year; ICH, Intracerebral Hemorrhage

**Supplementary Figure 1. Joinpoint analysis of SDI regions.**


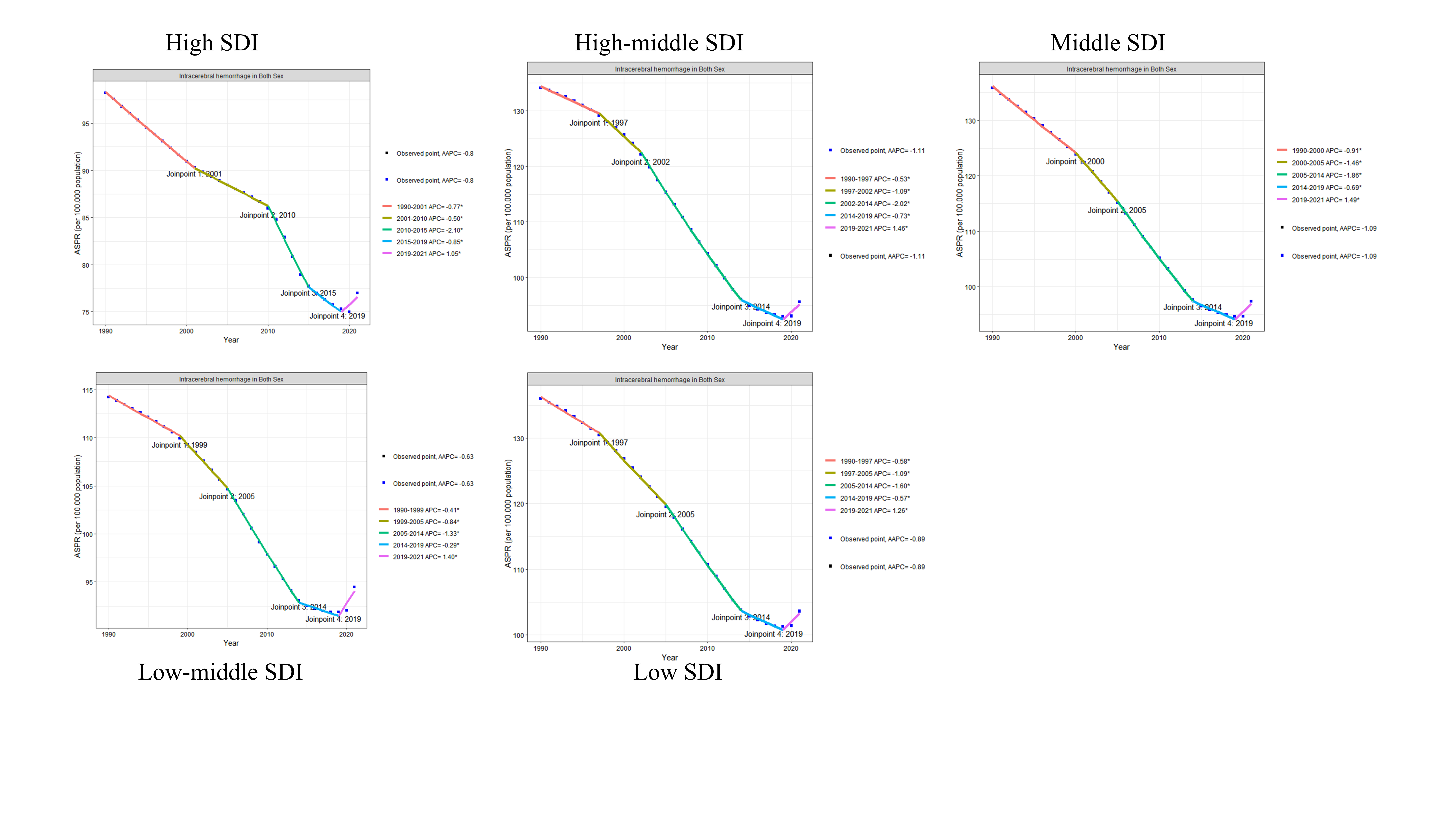

Supplement: Supplementary file 1 [file Data_Sheet_1.DOCX]
